# Bi-phasic effect of gelatin in myogenesis and skeletal muscle regeneration

**DOI:** 10.1242/dmm.049290

**Published:** 2021-12-24

**Authors:** Xiaoling Liu, Er Zu, Xinyu Chang, Xiaowei Ma, Ziqi Wang, Xintong Song, Xiangru Li, Qing Yu, Ken-ichiro Kamei, Toshihiko Hayashi, Kazunori Mizuno, Shunji Hattori, Hitomi Fujisaki, Takashi Ikejima, Dan Ohtan Wang

**Affiliations:** 1 Wuya College of Innovation, Shenyang Pharmaceutical University, Shenyang, 110016, China; 2 School of Traditional Chinese Materia Medica, Shenyang Pharmaceutical University, Shenyang 110016, China; 3 School of Life Science and Biopharmaceutic, Shenyang Pharmaceutical University, Shenyang 110016, China; 4 Institute for Integrated Cell-Material Science (iCeMS), Kyoto University, Yoshida-Honmachi, Sakyo-ku, Kyoto 606-850, Japan; 5 Department of Chemistry and Life Science, School of Advance Engineering, Kogakuin University, 2665-1, Nakanomachi, Hachioji, Tokyo 192-0015, Japan; 6 Nippi Research Institute of Biomatrix, Toride, Ibaraki 302-0017, Japan; 7 Key Laboratory of Computational Chemistry-Based Natural Antitumor Drug Research and Development, Shenyang Pharmaceutical University, Shenyang 110016, Liaoning, China; 8 Center for Biosystems Dynamics Research (BDR), RIKEN, 2-2-3 Minatojima-minamimachi, Chuo-ku, Kobe, Hyogo 650-0047, Japan

**Keywords:** Gelatin, Skeletal muscle regeneration, Hormesis, ROS, NOX2, IL-6/TNFα

## Abstract

Skeletal muscle regeneration requires extracellular matrix (ECM) remodeling, including an acute and transient breakdown of collagen that produces gelatin. Although the physiological function of this process is unclear, it has inspired the application of gelatin to injured skeletal muscle for a potential pro-regenerative effect. Here, we investigated a bi-phasic effect of gelatin in skeletal muscle regeneration, mediated by the hormetic effects of reactive oxygen species (ROS). Low-dose gelatin stimulated ROS production from NADPH oxidase 2 (NOX2) and simultaneously upregulated the antioxidant system for cellular defense, reminiscent of the adaptive compensatory process during mild stress. This response triggered the release of the myokine IL-6, which stimulates myogenesis and facilitates muscle regeneration. By contrast, high-dose gelatin stimulated ROS overproduction from NOX2 and the mitochondrial chain complex, and ROS accumulation by suppressing the antioxidant system, triggering the release of TNFα, which inhibits myogenesis and regeneration. Our results have revealed a bi-phasic role of gelatin in regulating skeletal muscle repair mediated by intracellular ROS, the antioxidant system and cytokine (IL-6 and TNFα) signaling.

## Abstract (precis)

**Summary:** Application of high- and low-dose gelatin to skeletal muscle revealed a bi-phasic role of gelatin in regulating skeletal muscle repair, which has translational implications for regenerative medicine.

## INTRODUCTION

Skeletal muscle has a remarkable capacity to regenerate after mechanical or disease-related injuries. This process often requires changes in the biological behavior of satellite cells (SCs), the myogenic progenitor cells, located within muscle extracellular matrix (ECM) (Ciciliot and Schiaffino, 2010; Chen and Shan, 2019). SCs are characterized by the expression of Pax7 and reside between the basal lamina and myofiber plasma membrane in resting skeletal muscle (Feng et al., 2018). Upon damage, quiescent SCs are rapidly activated to generate Pax7^+^/MyoD^+^ (also known as MYOD1) myoblasts that proliferate, migrate to the injury site, further differentiate into MyoD^+^/myogenin^+^ (MyoG^+^) myocytes, and fuse with each other or with damaged myofibers to generate myotubes and new multinucleated myofibers. The myogenesis process is under the control of myogenic regulatory factors (MRFs) – such as Myf5, Myf6, MyoD and MyoG – that direct myoblasts to enter myogenesis programs and further fuse with myofibers to repair muscle function. The myogenesis process is also signaled and aided by ECM remodeling, growth factors and myokines. The regenerative capability of skeletal muscle is life-long but susceptible to aging, neuromuscular diseases and ECM deficits (Joanisse et al., 2017).

Skeletal muscle is encapsulated by a well-organized network of connective tissue comprising ECM with embedded myoblasts, SCs and other cell types (Petrosino et al., 2019). Collagen (I, III, IV, XII, and XIV) is abundant in muscle ECM, accounting for nearly 10% of dry muscle weight. During skeletal muscle regeneration, collagen is digested by collagenase or degraded under inflammation or thermal condition into gelatin, which can be further hydrolyzed into smaller peptides by gelatinases such as metalloproteinase-2 and -9 (MMP-2 and MMP-9) (Bailey, 2000; Liu et al., 2018a; Chung et al., 2004). However, it is unknown whether gelatin influences the cellular behaviors of myoblasts during muscle repair. In recent years, gelatin as a biomaterial has been found to have wide applications in the food industry and tissue engineering due to its excellent biocompatibility, biodegradability and low cost (Nur Hanani et al., 2014). Gelatin-based biomaterials thus hold great potential in muscle tissue engineering. Ostrovidov et al. fabricated gelatin multi-walled carbon nanotubes to scaffold myotube formation and improve myotube contractions (Ostrovidov et al., 2014) and synthesized gelatin-polyaniline nanofibers to enhance the maturation of the excitation–contraction coupling system in myocytes (Ostrovidov et al., 2017). Gelatin–genipin-based hydrogels drive myogenic cell differentiation and can thus potentially be applied in skeletal muscle repair (Gattazzo et al., 2018). Although gelatin-based biomaterials are applied widely in tissue engineering, including muscle, their biological functions in myogenesis or muscle regeneration are unknown. In this study, we aimed to delineate the biological pathways of gelatin treatments in myogenesis and muscle regeneration for exploiting the potential benefits of gelatin application in treating skeletal muscle-related diseases and muscle regeneration.

Gelatin has previously been shown to induce reactive oxygen species (ROS) in multiple cell types to regulate various cell functions. ROS are major mediators of ECM remodeling during regeneration or ECM-related diseases. ROS and antioxidants are associated with skeletal muscle physiology and pathology, suggesting that gelatin may regulate muscle function through ROS signals. ROS in skeletal muscle primarily functions in redox signaling through mechanisms of hormesis, by which low-level exposure to ROS elicits beneficial stress adaptation, whereas elevated ROS production relative to the antioxidant capacity promotes oxidative stress and cytotoxicity. Interestingly, ROS signal has been reported to both enhance and inhibit myogenesis, depending on its concentration and location. The moderate production of ROS during exercise or regeneration induces myogenic differentiation of satellite cells and myoblasts, while excessive accumulation of ROS results in their senescence, apoptosis and regenerative failure in muscle repair (Le Moal et al., 2017). ROS are likely to function as a double-edged sword in myogenesis. It has been reported that the function of ROS in myogenesis is mediated by inflammatory factors, such as NF-κB p65 (also known as RELA) and FOXO1 (Dodd et al., 2010). However, the cellular and molecular mechanisms underlying the dual effects of ROS on myogenesis are not fully understood.

Herein we have investigated gelatin-induced biological effects on skeletal muscle repair and intracellular signaling pathways in C2C12 cells, primary myoblasts and tibialis anterior (TA) muscle to elucidate the cellular mechanisms underlying the functional role of gelatin during myogenesis and muscle regeneration. Our results have revealed a bi-phasic role of gelatin in regulating skeletal muscle repair mediated by intracellular ROS, the antioxidant system, and cytokine [IL-6 and TNFα (also known as TNF)] signaling cascades.

## RESULTS

### Bi-phasic role of gelatin in skeletal muscle regeneration *in vivo*

**Fig. 1.**
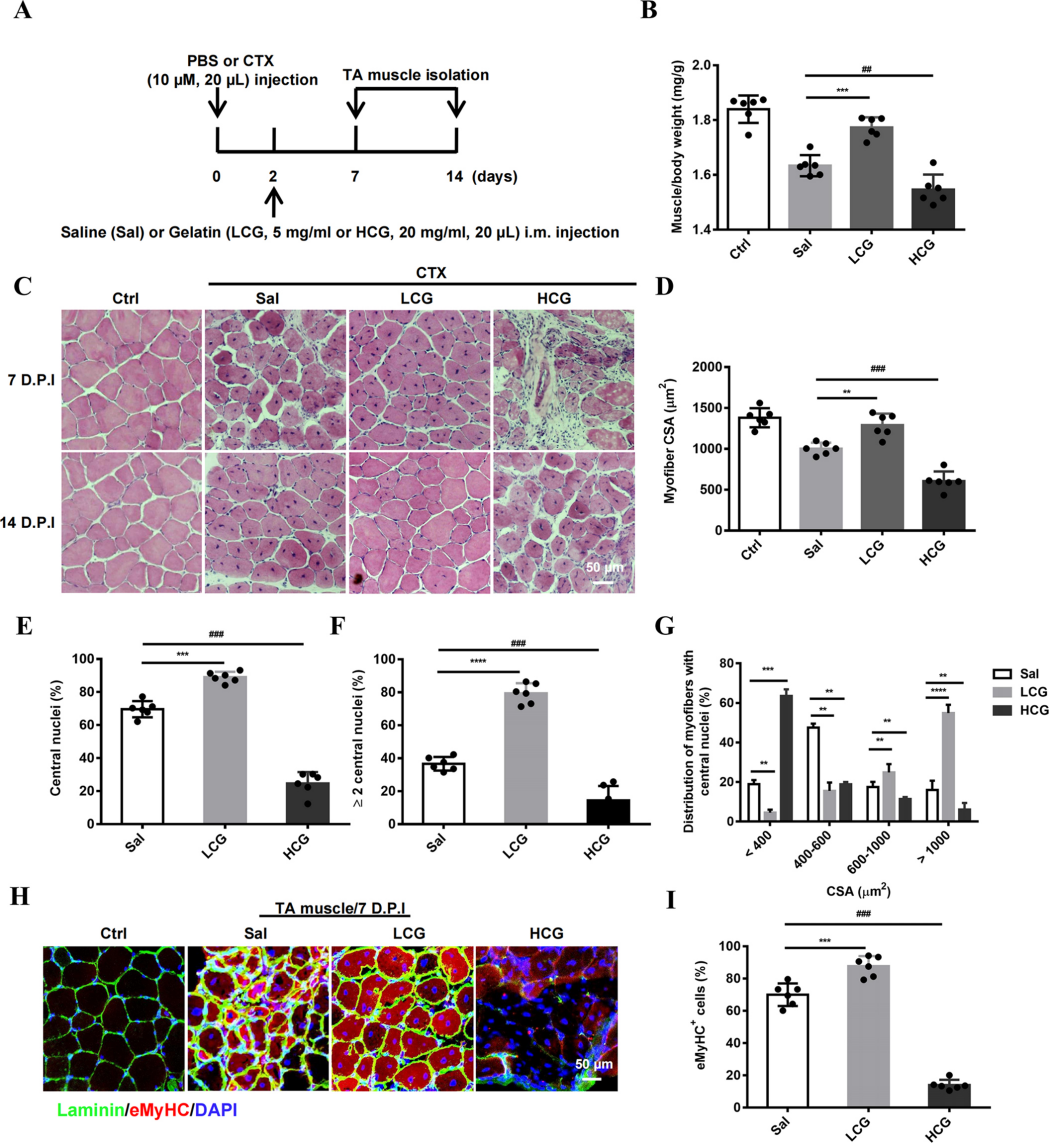
**Bi-phasic effect of gelatin in muscle regeneration *in vivo*.** (A) Experimental schematic diagram depicting cardiotoxin (CTX) intramuscular (i.m.) injection to induce muscle injury (10 μM, 20 μl in PBS) and gelatin injection (LCG, 5 mg/ml; HCG, 20 mg/ml, diluted with saline) 2 days post-injury (D.P.I.). The tibialis anterior (TA) muscles were collected at 7 and 14 D.P.I. PBS-injected animals were uninjured (Ctrl). (B) TA mass normalized to animal body mass (*n*=6). (C) H&E staining of the damaged TA muscle sections at 7 (top row) and 14 (bottom row) D.P.I. Scale bar: 50 μm. (D) Average cross-sectional areas (CSA) of regenerated myofibers (*n*=6). (E) The percentage of myofibers containing central nuclei (*n*=6). (F) The percentage of myofibers with two or more central nuclei at 14 D.P.I. (*n*=6). (G) CSA distribution of myofibers at 14 D.P.I. (*n*=6). (H) Confocal images of myofibers (green, laminin; red, eMyHC; blue, DAPI) in damaged TA muscle sections at 7 D.P.I. Scale bar: 50 μm. (I) The percentage area occupied by eMyHC^+^ myofibers (*n*=6). Significance was determined by unpaired two-tailed Student's *t*-test with Welch's correction. ^##,^**P<0.01; ^###,^***P<0.001; ****P<0.0001. Data are mean±s.e.m. HCG, high concentration of gelatin; LCG, low concentration of gelatin.

To investigate the function of gelatin in skeletal muscle repair, we injected low (LCG; 20 µl of 5 mg/ml) and high (HCG; 20 µl of 20 mg/ml) concentrations of gelatin (saline as control) into cardiotoxin (CTX)-damaged TA muscle 2 days after injury. Recovery was first evaluated at 7 and 14 days post-injury (D.P.I.) by weighing muscle mass and histological examination (Fig. 1A). Compared to saline-injected control mice, LCG-injected mice recovered significantly more TA muscle mass at 14 D.P.I., albeit not as much as in uninjured muscle (Fig. S1A,B). Remarkably, HCG not only did not facilitate recovery but aggravated the original injury, resulting in further muscle loss (Fig. 1B). Hematoxylin and Eosin (H&E) and immunohistochemical (IHC) staining also revealed that, at 7 D.P.I., LCG mice formed a higher number of new muscle fibers, characterized by more centrally located nuclei than in control mice, whereas little muscle fiber regeneration was observed in HCG mice. At 14 D.P.I., LCG mice displayed tightly packed, well-formed muscle fibers resembling uninjured muscle in control (Ctrl) mice, but HCG mice had smaller muscle fibers with single nuclei, indicating incomplete regeneration and thus impeded progress compared to natural recovery (Fig. 1C; whole-section view in Fig. S1C). We quantified the cross-sectional area (CSA) of muscle fibers, the number of nuclei and the percentage of myofibers containing central nuclei. These parameters consistently showed facilitated recovery in LCG mice and impeded recovery in HCG mice compared to the saline-treated group, indicating a bi-phasic effect that, at lower concentrations of gelatin injection, can aid muscle regeneration, but at higher concentrations can obstruct muscle regeneration (Fig. 1D-G). We further stained embryonic myosin heavy chain (eMyHC; also known as MYH3), a skeletal muscle-specific contractile protein expressed during muscle development. Co-staining of eMyHC and laminin revealed that the number of eMyHC^+^/laminin^+^ myofibers was significantly increased by LCG but reduced by HCG (Fig. 1H,I). This result further supported that gelatin can facilitate muscle regeneration in a bi-phasic and concentration-dependent manner.

### Low-dose gelatin promotes SC expansion and myogenic differentiation

**Fig. 2.**
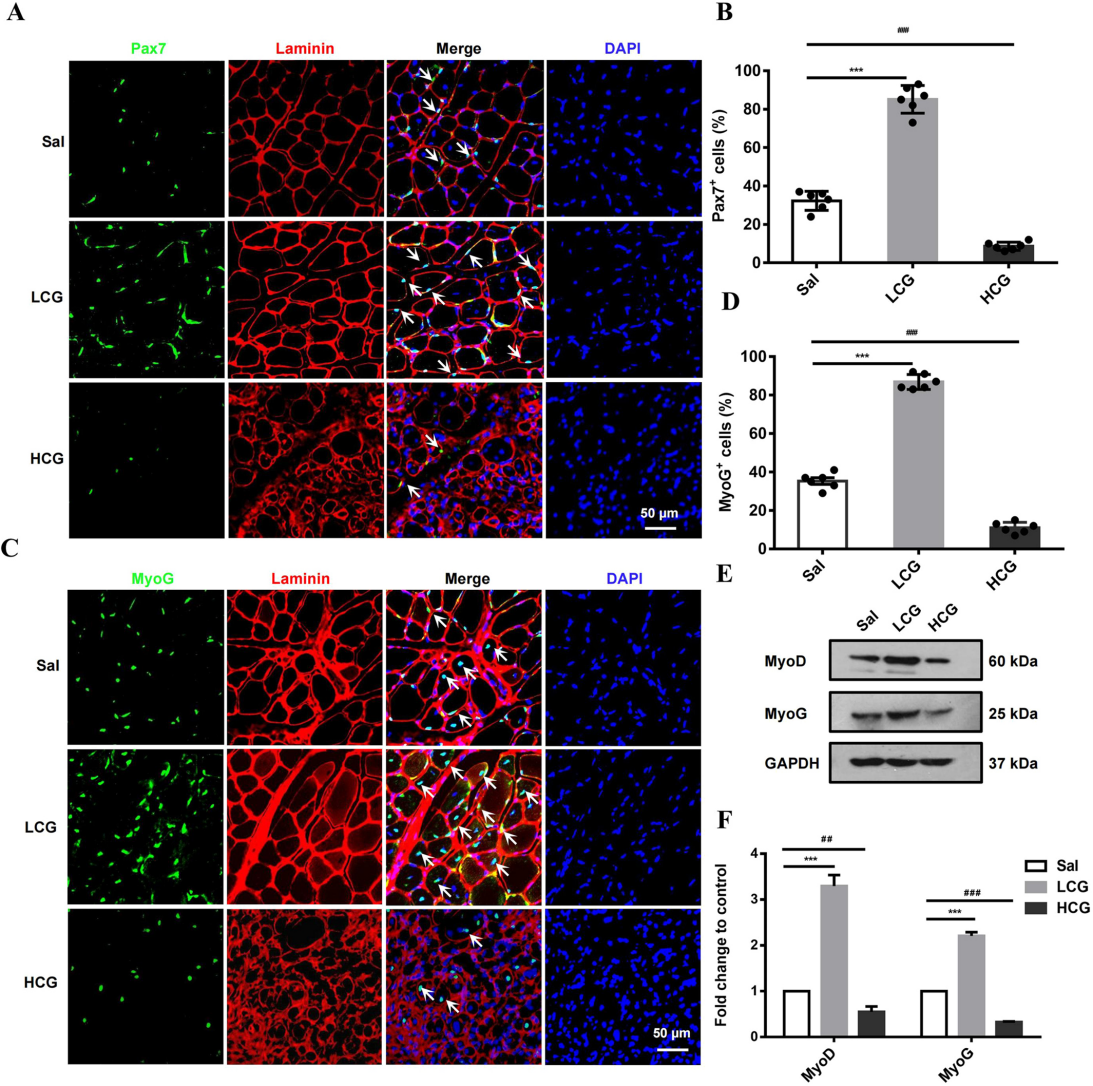
**Bi-phasic effect of gelatin in activating quiescent satellite cells (SCs) and myogenesis during muscle regeneration.** (A) Immunohistochemical (IHC) analysis of Pax7^+^ SCs (green) in TA muscle stained by laminin (red) at 7 D.P.I. Arrows point to Pax7^+^ nuclei. Scale bar: 50 μm. (B) The percentage of myofibers containing Pax7^+^ SCs (*n*=6). (C) IHC analysis of MyoG^+^ cells (green) in damaged TA muscle stained by laminin (red) at 7 D.P.I. Arrows point to MyoG^+^ nuclei. Scale bar: 50 μm. (D) The percentage of myofibers containing MyoG^+^ cells (*n*=6). (E,F) Western blots (E) and quantification (F) of MyoD and MyoG in damaged TA muscles at 14 D.P.I. (*n*=3). GAPDH is used as a loading control. Significance was determined by unpaired two-tailed Student's *t*-test with Welch's correction. ^##^*P*<0.01; ^###,^***P<0.001. Data are mean±s.e.m.

Activation of SCs and myogenic differentiation are key steps in skeletal muscle repair. Co-staining of Pax7 and laminin in TA muscle indicated expansion of SCs during muscle regeneration. More Pax7^+^ SCs located underneath the basal lamina were detected at 7 D.P.I. after LCG injection and less after HCG injection compared with the saline group (Fig. 2A,B). Furthermore, fluorescence co-staining of MyoG and laminin revealed that LCG increased, but HCG reduced, the number of MyoG^+^/laminin^+^ cells in the damaged muscle sites (Fig. 2C,D). Consistently with the immunostaining results, western blots showed up- and downregulated expression of MyoD and MyoG by LCG and HCG, respectively (Fig. 2E,F). Taken together, these results indicate that, during *in vivo* TA muscle regeneration, injecting gelatin into the injury site can promote SC expansion, myoblast differentiation and fusion, but only when injected at a lower amount, such as 20 µl of 5 mg/ml.

### Gelatin influences myoblast behavior in C2C12 myoblasts

**Fig. 3.**
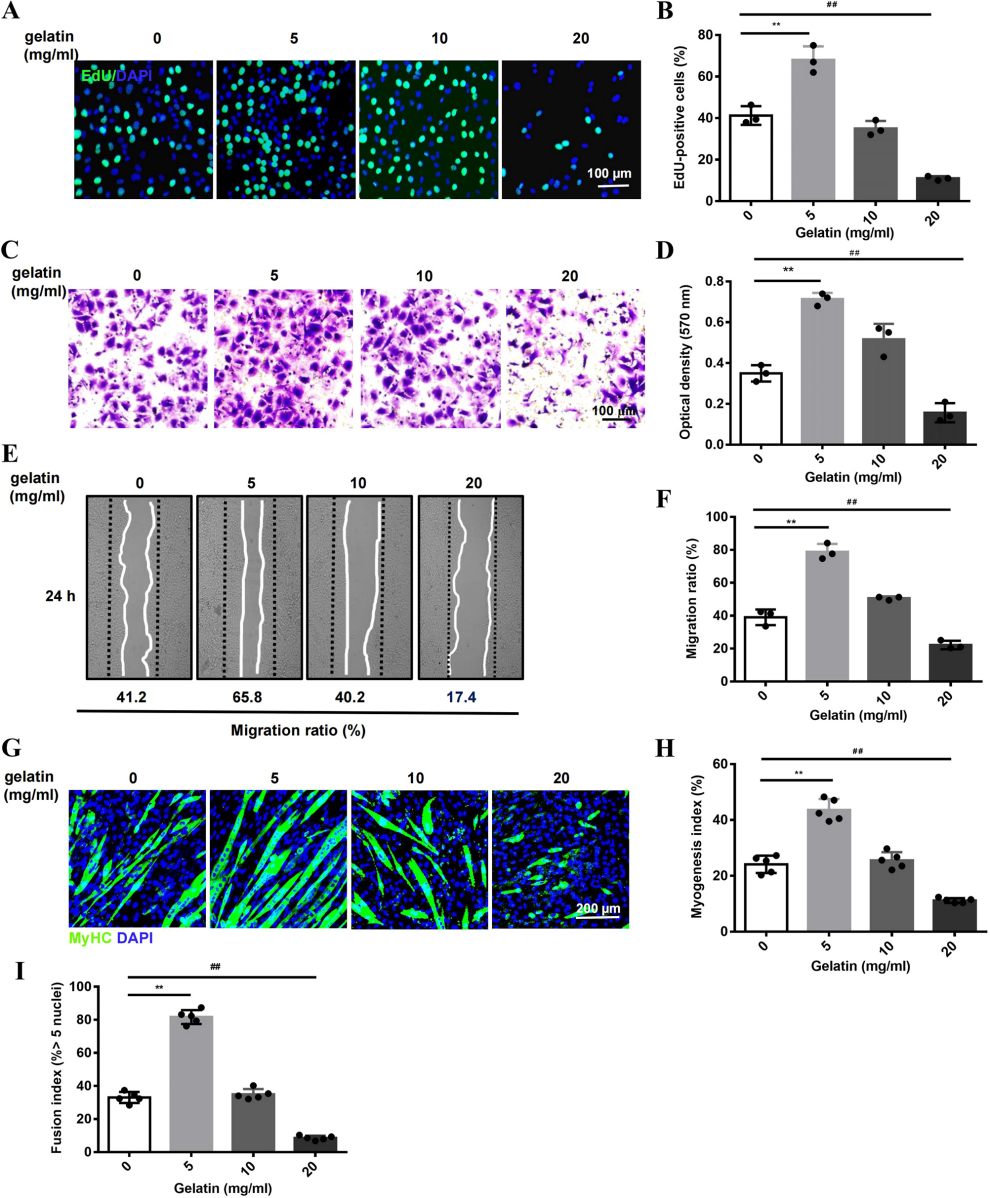
**Bell-shaped response of myogenesis to gelatin *in vitro*.** (A) Confocal images of C2C12 cells on gelatin-coated dishes, stained with Hoechst (blue) and EdU (green). Scale bar: 100 μm. (B) Quantification of EdU^+^ (proliferating) cells (*n*=3). (C,D) Transwell assay: Crystal Violet staining images (C) and quantification (D) (*n*=3). Scale bar: 100 μm. (E,F) Wound healing assay: cell images (E) and quantification (F) (*n*=3). (G) Confocal images of myogenic differentiation of cells stained with an anti-MyHC antibody (Ab). Scale bar: 200 μm. (H) Myogenesis index (percentage of nuclei within MyHC-stained myocytes/total nuclei) (*n*=5). (I) Fusion index (percentage of nuclei in myotubes with >5 nuclei/total nuclei in MyHC-stained cells) (*n*=5). Significance was determined by unpaired two-tailed Student's *t*-test with Welch's correction. ^##,^**P<0.01. Data are mean±s.e.m.

To elucidate the cellular mechanisms underlying the bi-phasic effect of gelatin, we tested the proliferation, migration and myogenic differentiation of C2C12 myoblasts cultured on gelatin-coated dishes (0, 5, 10, 20 mg/ml). The proliferation rate was measured using 5-ethynyl-2′-deoxyuridine (EdU) labeling. After 24 h incubation, cell numbers showed a bell-shaped response to gelatin, highest at 5 mg/ml (LCG) and lowest at 20 mg/ml (HCG) (Fig. 3A,B). Compared to the cells cultured on non-coated dishes or dishes coated in gelatin at 10 mg/ml, which were large and with a flattened shape, the cells cultured on LCG appeared extended, bipolar and fibroblast-like, while the cells cultured on HCG appeared irregular, multi-polar and satellite-like (Fig. S2A). Furthermore, the migration of cells was enhanced nearly twofold on LCG dishes but inhibited on HCG dishes (Fig. 3C-F). For myogenic differentiation, LCG not only increased the mRNA expression of myogenic factors, including *Myod*, *Myog* and myosin heavy chain (MyHC; *Myh1*) (Fig. S2B), but also increased the number of MyHC^+^ cells (myogenesis index) and promoted the formation of myotubes (fusion index) (Fig. 3G-I) characterized by a wider diameter and longer length compared with those of control cells (Fig. S2C,D). By contrast, HCG decreased the myogenic differentiation of cells (Fig. 3G-I). Thus, the bi-phasic effect of gelatin on myogenesis *in vivo* is reproduced in C2C12 myoblast cells *in vitro*.

### Gelatin enhances ROS generation in a dose-dependent manner

**Fig. 4.**
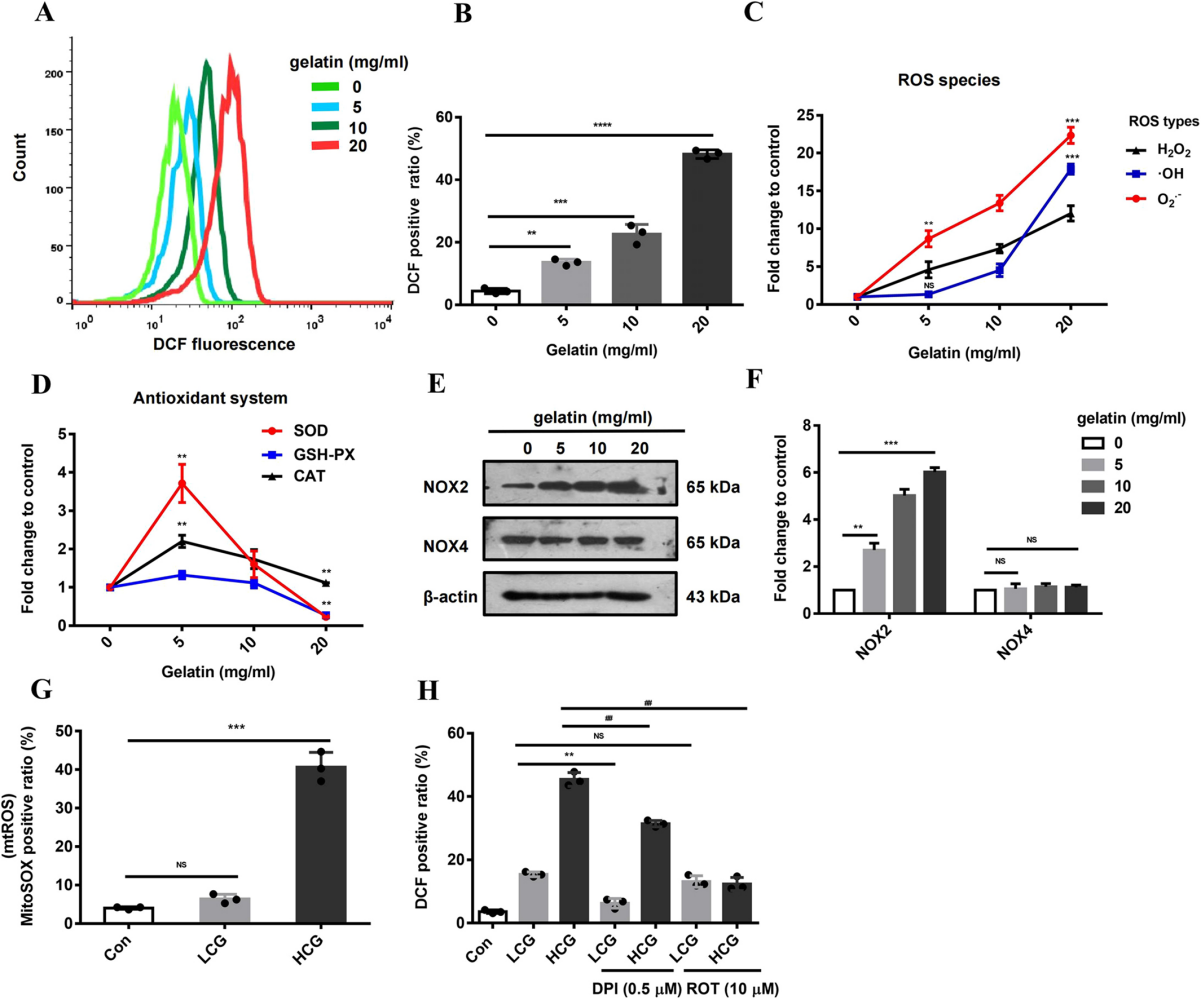
**Linear and bell-shaped dose response of reactive oxygen species (ROS) and the antioxidant system to gelatin.** (A,B) Flow cytometry analysis (A) and quantification (B) of cells stained with 2′,7′-dichlorodihydrofluorescein diacetate (DCFH-DA; DCF) (ROS indicator). Count (*y*-axis in A) indicates cell counts (*n*=3). (C) Production of specific ROS: O_2_^−^, ·OH and H_2_O_2_ (*n*=3). (D) Activities of antioxidases: superoxide dismutase (SOD), glutathione peroxidase (GSH-PX) and catalase (CAT) (*n*=3). (E,F) Western blots (E) and quantification (F) of NOX2 and NOX4 in the cells cultured on gelatin-coated dishes at 90% confluence (*n*=3). β-Actin is used as a loading control. (G) Quantification of MitoSOX^+^ cells obtained by flow cytometry (*n*=3). (H) Flow cytometry analysis of the cells treated with diphenyliodonium (DPI; 0.5 μM, NOX2 inhibitor) and rotenone (ROT; 10 μM, an inhibitor of mitochondrial respiratory complex I) (*n*=3). Significance was determined by unpaired two-tailed Student's *t*-test with Welch's correction (B-D,F-G) and one-way ANOVA with Tukey's post-hoc test (H). ^##,^**P<0.01; ***P<0.001; ****P<0.0001; NS, not significant. Data are mean±s.e.m.

ROS have been shown to function as a double-edged sword in multiple cell functions and downstream cellular signaling of gelatin (Chen et al., 2019; Liu et al., 2018b). Using 2′,7′-dichlorodihydrofluorescein diacetate (DCFH-DA) staining (an indicator of ROS), we found that gelatin stimulated ROS production in C2C12 cells in a dose-dependent manner (Fig. 4A,B). The cellular concentrations of ROS such as superoxide radical (O_2_^−^), hydroxyl radical (·OH) and hydrogen peroxide (H_2_O_2_) all responded to gelatin coating. LCG significantly enhanced the production of O_2_^−^; HCG dramatically increased O_2_^−^ and also ·OH (Fig. 4C). We further tested the activity of the antioxidant system consisting of antioxidases, including superoxide dismutase (SOD), glutathione peroxidase (GSH-PX) and catalase (CAT), together with non-enzymatic antioxidants (He et al., 2017). The results indicated that the activity of antioxidases, especially SOD, was increased by LCG, but gradually inhibited by increasing concentrations of gelatin. HCG significantly suppressed the activity of GSH-PX and SOD, explaining the accumulation of ROS (Fig. 4D).

We then sought to identify the cellular source for gelatin-induced ROS production. ROS can be produced by multiple sources, including NADPH oxidases (NOXs) and the mitochondrial electron transport complex. NOXs are a vital enzyme family that catalyzes ROS production, of which NOX2 (also known as CYBB) and NOX4 are the main isoforms found in skeletal muscle. It is known that NOXs participate in skeletal muscle metabolism and function through redox regulation (Henriquez-Olguin et al., 2019). Western blots showed that expression of NOX2, but not NOX4, was upregulated by gelatin in a dose-dependent manner (Fig. 4E-F). We next examined O_2_^−^ and ·OH produced in mitochondria, another significant source of ROS production from oxygen consumption. MitoSOX Red, a selective fluorescence dye for mitochondrial ROS, was used in flow cytometry analysis (Fig. 4G). Results showed that HCG specifically promoted the generation of mitochondrial ROS, indicating that multiple cellular sources of ROS production can be induced by gelatin. To verify the above findings, we applied specific inhibitors of NOX2, and of complex I of the mitochondrial respiratory chain, diphenyliodonium (DPI) and rotenone (ROT), respectively, to C2C12 cells cultured on LCG and HCG-coated dishes. DCFH-DA staining and flow cytometry analysis showed that DPI (0.5 μM) treatment suppressed ROS production in both LCG and HCG groups, whereas ROT (10 μM) treatment specifically blocked ROS overproduction by HCG (Fig. 4H). Together with ROS measurements, these results indicate that LCG primarily induces production of O_2_^−^ by NOX2; HCG not only further upregulates NOX2, but also stimulates the production of O_2_^−^ and ·OH by mitochondrial respiratory complex I. The markedly high level of ROS under HCG conditions may also be partially attributed to the inhibition of antioxidant activities of SOD and GSH-PX.

### The hormetic function of ROS in myoblast cellular behaviors

**Fig. 5.**
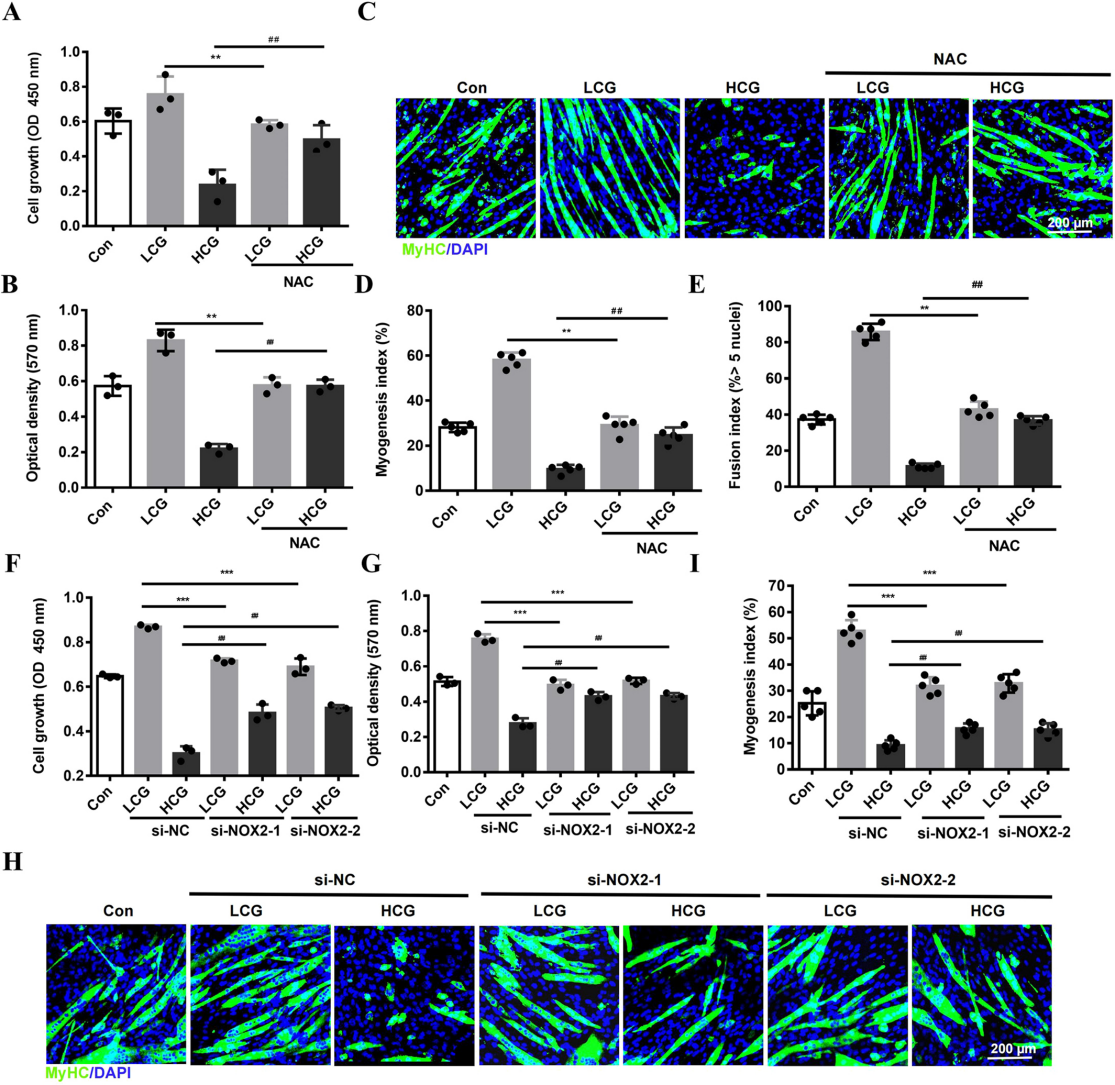
**ROS signaling is required for the bi-phasic effect of gelatin.** (A,B) Growth (A) and migration (B) of C2C12 cells treated with N-acetyl-L-cysteine (NAC), an ROS scavenger (8 mM) (*n*=3). OD, optical density. (C) Confocal images of myogenic differentiation of cells stained with anti-MyHC Ab. Scale bar: 200 μm. (D,E) Myogenesis index (percentage of nuclei within MyHC-stained myocytes/total nuclei; D) and fusion index (percentage of nuclei in myotubes with >5 nuclei/total nuclei in MyHC-stained cells; E) (*n*=5). (F,G) Growth (F) and migration (G) of cells after silencing *Nox2* expression (si-NOX2-1, si-NOX2-2 or si-NC as negative control) (*n*=3). (H) Confocal images of myogenic differentiation of cells stained with anti-MyHC Ab. Scale bar: 200 μm. (I) Myogenesis index (*n*=5). Significance was determined by one-way ANOVA analysis with Tukey's post-hoc test.^##,^**P<0.01; ^###,^***P<0.001. Data are mean±s.e.m.

To understand the effects of ROS on myoblast behaviors, different concentrations of tertiary butylhydroperoxide (*t*BHP), a donor of O_2_^−^ and ·OH, were added to the culture medium. Previous studies revealed that a low dose of *t*BHP (lower than 25 μM) moderately promoted the levels of ROS, enhancing the proliferation and migration of 3T3-L1 fibroblast cells, whereas high *t*BHP concentration (50 μM) caused detrimental accumulation of ROS that inhibited cell behaviors (Liu et al., 2018b). In this study, we added *t*BHP (5, 10, 50 μM) to C2C12 cell culture and confirmed dose-dependent induction of ROS using flow cytometry analysis (Fig. S3A). At 10 μM, *t*BHP promoted the growth, migration and myogenic differentiation of C2C12 cells, but at 50 μM, *t*BHP had the opposite effects, suggesting a hormetic effect of ROS (Fig. S3B-H). Next, N-acetyl-L-cysteine (NAC), a scavenger of ROS, was added to the cultures. Removal of ROS was confirmed using flow cytometry (Fig. S4A). NAC treatment diminished LCG and HCG effects on cell proliferation (Fig. 5A; Fig. S4B) and cell migration (Fig. 5B). Similarly, NAC reduced both the beneficial effect of LCG and the deleterious effect of HCG in regulating the genesis of MyHC^+^ cells and fusion of myotubes (Fig. 5C-E; Fig. S4C,D). The results were further supported by the expression levels of mRNAs and proteins of myogenic factors, MyoD, MyoG and MyHC (Fig. S4E-G), thus directly demonstrating a mechanism of hormesis through ROS generation that underlies gelatin's effect on myogenesis.

To verify the role of NOX2 in mediating gelatin’s effects, we knocked down *Nox2* using siRNA, the efficiency of which was confirmed by reduced expression of NOX2 and lowered levels of ROS production in LCG and HCG (Fig. S4H-J). *Nox2* silencing reduced both LCG and HCG effects on cell growth and migration (Fig. 5F,G). Downregulation of NOX2 in LCG-treated cells also decreased the number of MyHC^+^ cells and the formation of myotubes indicated by the myogenesis index (Fig. 5H,I). The myotubes showed narrower width and shorter length compared with cells transfected with si-NC (negative control) (Fig. S4K,L). By contrast, the transfection of *Nox2* siRNA increased the myogenic differentiation of cells on HCG (Fig. 5H,I). These data indicated that NOX2 is an important upstream factor in the myoblast to mediate gelatin’s effects.

### IL-6 and TNFα mediate hormetic ROS function for regulating myogenesis

**Fig. 6.**
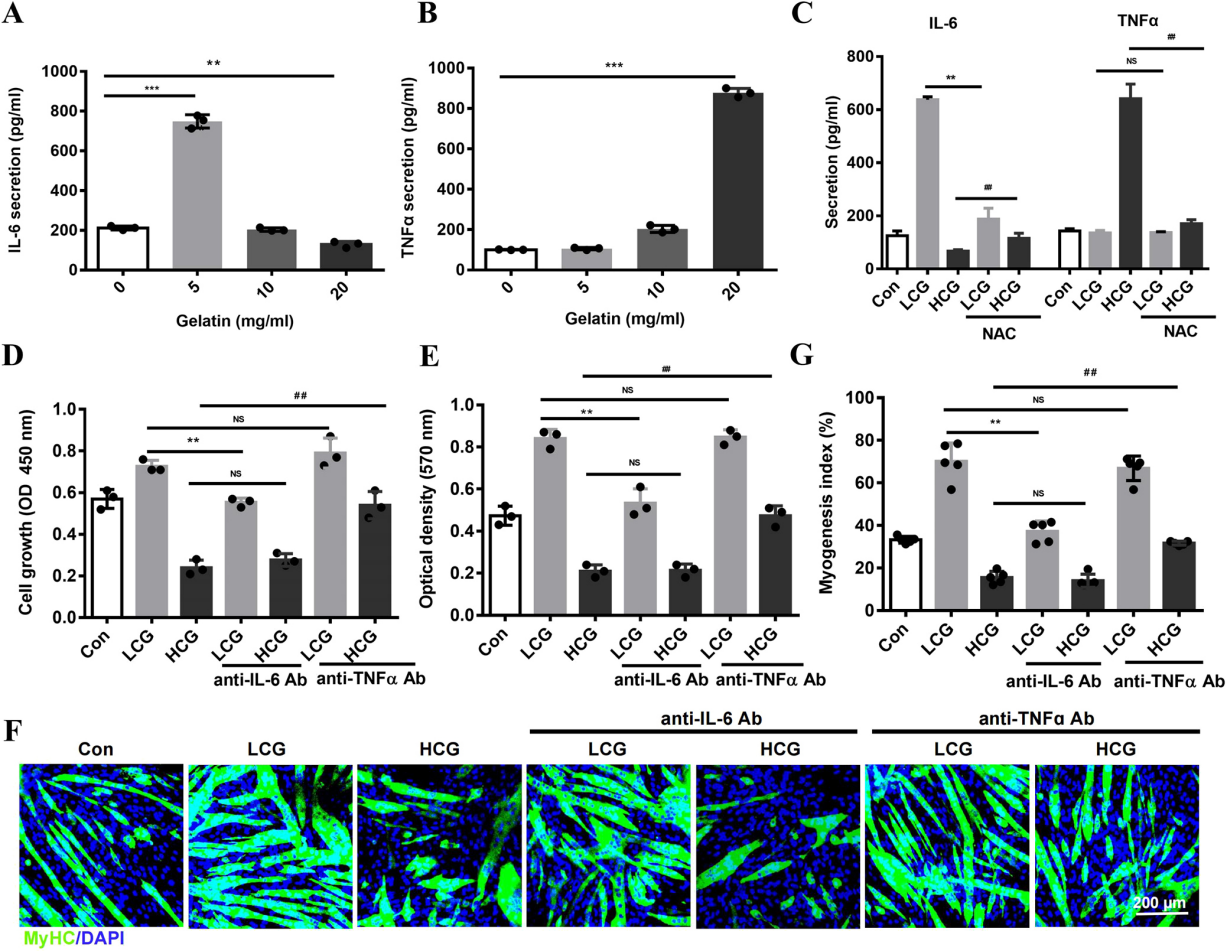
**IL-6 and TNFα mediate dual regulation of ROS in myogenesis.** (A,B) Enzyme-linked immunosorbent assay (ELISA) of IL-6 (A) and TNFα (B) in the supernatants of C2C12 cells cultured on gelatin (*n*=3). (C) ELISA of IL-6 and TNFα in the culture supernatants of C2C12 cells treated with NAC (*n*=3). (D,E) Growth (D) and migration (E) of C2C12 myoblasts incubated with the neutralizing Abs against IL-6 (0.3 μg/ml) or TNFα (0.5 μg/ml) (*n*=3). (F) Confocal images of myogenic differentiation of cells stained with anti-MyHC Ab (green) and DAPI (blue) after incubation with anti-IL-6 and anti-TNFα. Scale bar: 200 μm. (G) Myogenesis index (*n*=5). Significance was determined by unpaired two-tailed Student's *t*-test with Welch's correction (A,B) and one-way ANOVA with Tukey's post-hoc test (C-E,G). ^##,^**P<0.01; ***P<0.001; NS, not significant. Data are means±s.e.m.

Myokines produced and released by skeletal muscle influence important biological functions of myocytes. They are known to be induced by ROS during physical exercise and muscle regeneration (Scheele et al., 2009). To identify specific myokines that mediate the effects of gelatin and ROS, we screened potential targets and identified IL-6, the secretion of which was promoted by LCG but slightly inhibited by HCG (Fig. 6A). In contrast to IL-6, the release of TNFα was specifically induced by HCG (Fig. 6B). Other myokines, such as the pro-inflammatory factors IL-1β and IL-18, as well as the chemokines CCL5 and MCP-1 (also known as CCL2), were unaffected by different doses of gelatin (Fig. S5A-D). To determine the role of ROS in myokine release, we added ROS donor *t*BHP to C2C12 cultures at different concentrations, which induced the release of IL-6 and TNFα in a parallel manner with gelatin (Fig. S5E). Furthermore, treatment of NAC (ROS scavenger) specifically blocked the release of IL-6 from LCG myoblasts and TNFα from HCG myoblasts, indicating that ROS are responsible for the activation of both myokine pathways (Fig. 6C).

To directly confirm the function of IL-6 and TNFα in gelatin-induced myogenesis, purified mouse recombinant myokines were exogenously added to C2C12 cultures. The results showed that IL-6 enhanced the growth and migration of cells and increased the number of wider and longer MyHC^+^ cells, whereas TNFα exerted an opposite effect (Fig. S6A-F). Then, we investigated whether the release of IL-6 and TNFα is necessary for the gelatin effect by adding IL-6- and TNFα-neutralizing monoclonal antibodies (Abs) to the culture medium. The enhanced growth and migration of cells on LCG were decreased by anti-IL-6 Ab but not by anti-TNFα Ab (Fig. 6D,E). By contrast, the inhibitory effect by HCG was reversed by anti-TNFα Ab but not by anti-IL-6 Ab (Fig. 6D,E). The blockade of IL-6 effectively decreased the number of MyHC^+^ cells (Fig. 6F,G), which resulted in the formation of narrower and shorter myotubes. By contrast, anti-TNFα Ab had no effect on LCG cells, but significantly reversed the HCG effect on myogenesis (Fig. S6G,H). These data reveal that the bi-phasic effect of gelatin on myogenesis can be mediated by IL-6 and TNFα, induced by low and high levels of ROS, respectively.

### Gelatin-induced myogenesis in mouse primary myoblasts

**Fig. 7.**
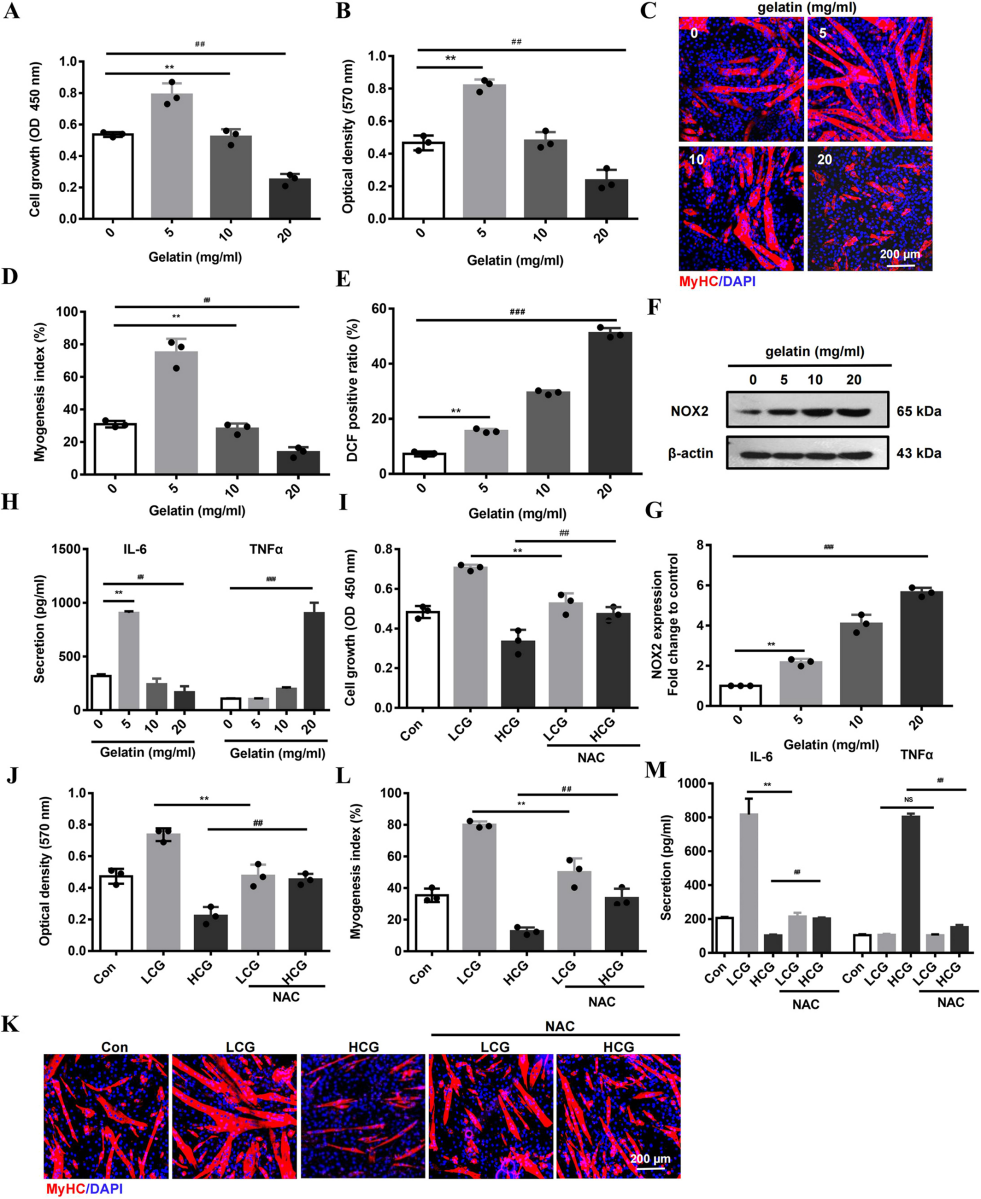
**ROS and cytokines mediate the bi-phasic effect of gelatin substrate in mouse primary myoblast (MPM) myogenesis.** (A,B) Growth (A) and migration (B) of MPMs show bell-shaped concentration response to gelatin substrates. (C) Confocal images of differentiated MPMs stained with anti-MyHC Ab (red) and DAPI (blue). Scale bar: 200 μm. (D) Myogenesis index. (E) Flow cytometry analysis of MPMs stained with DCFH-DA. (F,G) Western blots (F) and quantification (G) of NOX2 proteins in MPMs cultured on increasing concentrations of gelatin substrate. β-Actin is used as a loading control. (H) ELISA of IL-6 and TNFα levels in the culture supernatants of MPMs. (I,J) Growth (I) and migration (J) of MPMs treated with NAC, an ROS scavenger (8 mM). (K) Confocal images of differentiated MPMs stained with anti-MyHC Ab (red) and DAPI (blue). Scale bar: 200 μm. (L) Myogenesis index. (M) ELISA of IL-6 and TNFα in the culture supernatants of MPMs treated with NAC (8 mM). Significance was determined by unpaired two-tailed Student's *t*-test with Welch's correction (A,B,D,E,G,H) and one-way ANOVA with Tukey's post-hoc test (I,J,L,M). ^##,^**P<0.01; ^###^*P*<0.001; NS, not significant. *n*=3. Data are mean±s.e.m.

In order to verify the gelatin–ROS–IL-6/TNFα signaling cascade in myogenesis that we have identified in C2C12 cells, we repeated experiments in mouse primary myoblasts (MPMs) isolated from TA muscle (Gharaibeh et al., 2008). The primary TA muscle cells adhered to the plates with a round morphology within 4 h after plating and developed flat fibroblast-like morphology, with Desmin (myoblast marker) detectable in 97% of the cell population (Fig. S7A-C). After reaching 90% confluence, cells were exposed to 2% horse serum to induce myogenic differentiation (Fig. S7A). Consistent with the results in C2C12 cells, the growth and migration of MPMs were enhanced by LCG but inhibited by HCG (Fig. 7A,B). LCG increased the number of MyHC^+^ cells (Fig. 7C,D) and promoted the formation of wider and longer myotubes compared with those of the control, whereas HCG exerted opposite effects (Fig. S7D-F). ROS production and NOX2 expression in MPMs were induced by gelatin in a similar manner to that in C2C12 cells (Fig. 7E-G). Removal of ROS by NAC (Fig. S7G) not only decreased the LCG-enhanced growth and migration of cells but also reversed the inhibitory effect of HCG on cell proliferation and migration (Fig. 7I,J). NAC also decreased the number of MyHC^+^ cells (Fig. 7K,L), myotube formation, and length and width of myotubes in the LCG group, while increasing myogenesis in the HCG cells (Fig. S7H-J). LCG significantly promoted IL-6 production. HCG slightly inhibited IL-6 but significantly enhanced TNFα secretion (Fig. 7H). The suppression of ROS by NAC reduced IL-6 and TNFα in culture medium in TA muscle myoblasts cultured on LCG and HCG substrates (Fig. 7M). These data suggested that the signaling cascade of gelatin–ROS–IL-6/TNFα underlying the bi-phasic effect of gelatin is valid in primary muscle cells.

### The gelatin–ROS–IL-6/TNFα cascade underlies skeletal muscle regeneration *in vivo*

**Fig. 8.**
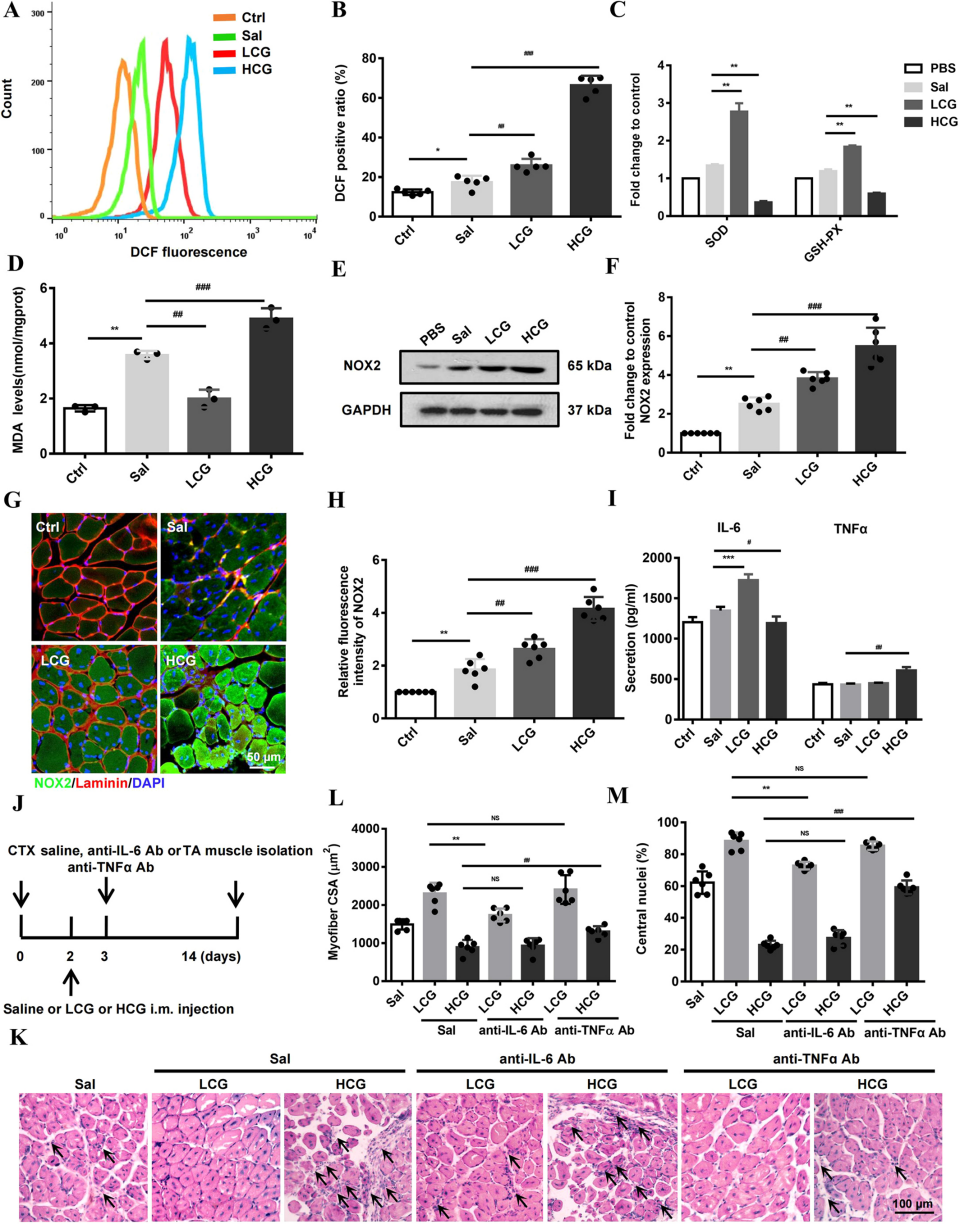
**ROS, antioxidant and cytokine signaling cascade upon low- and high-dose gelatin injection during muscle regeneration.** (A,B) Flow cytometry analysis (A) and quantification (B) of ROS in damaged TA muscle cells stained with DCFH-DA (*n*=5). Count (*y*-axis in A) indicates cell counts. (C) The activities of SOD and GSH-PX in damaged TA muscle (*n*=3). (D) Malondialdehyde (MDA) level in the damaged TA muscle cells (*n*=3). (E,F) Western blots (E) and quantification (F) of NOX2 in the damaged TA muscle (*n*=6, GAPDH as a loading control). (G) IHC analysis of NOX2 (green) in the damaged TA muscles stained with laminin (red) and DAPI (blue) at 7 D.P.I. Scale bar: 50 μm. (H) Fluorescence intensity of NOX2 relative to uninjured myofibers (*n*=6). (I) ELISA of IL-6 and TNFα in damaged TA muscle tissue at 7 D.P.I. (*n*=3). (J) Experimental schematic diagram showing saline, anti-IL-6 Ab (0.25 mg/kg) and anti-TNFα Ab (0.5 mg/kg) intramuscular injection 3 days after CTX injury. The TA muscles were collected at 14 D.P.I. (K) H&E staining of the damaged TA muscle sections in anti-IL-6 Ab- and anti-TNFα Ab-treated mice at 14 D.P.I. (*n*=6). Scale bar: 100 μm. Arrows indicate inflammatory infiltration. (L) CSA of regenerated myofibers (*n*=6). (M) Percentage of myofibers containing central nuclei (n=6). Significance was determined by unpaired two-tailed Student's *t*-test with Welch's correction (C,I) and one-way ANOVA with Tukey's post-hoc test (B,D,F,H,L,M). ^#,^*P<0.05; ^##,^**P<0.01; ^###,^***P<0.001. Data are mean±s.e.m.

Finally, we examined the oxidative status in the gelatin-injected TA muscle at 14 D.P.I. DCFH-DA staining showed that LCG modestly promoted ROS generation compared with saline, and HCG further aggravated the accumulation of ROS (Fig. S8A,B). Similar results were also obtained from the analysis of DCFH-DA staining of TA muscle cells examined by flow cytometry (Fig. 8A,B). The results showed that ROS levels, as well as the antioxidant activities of SOD and GSH-PX, were upregulated modestly by LCG. Furthermore, high accumulation of ROS, and reduced activities of SOD and GSH-PX, were observed in TA muscle of HCG injection (Fig. 8A-C). The overaccumulation of ROS by HCG injection also caused a higher level of malondialdehyde (MDA) detected in the regenerated muscle, indicating higher oxidative stress, whereas LCG reduced MDA (Fig. 8D). We further examined NOX2 expression and found that the protein was not only upregulated during natural muscle regeneration but was further increased by gelatin injection in a dose-dependent manner (Fig. 8E-H). Then, we examined the levels of IL-6 and TNFα in 14 D.P.I. TA muscles. The results showed an increase in IL-6 in the regenerating muscle, which was further enhanced by LCG but slightly inhibited by HCG. By contrast, TNFα induction was detected specifically in HCG animals (Fig. 8I). Lastly, we injected anti-IL-6 Ab (0.25 mg/kg) or anti-TNFα Ab (0.5 mg/kg) into the injury site 1 day after LCG or HCG injection (Fig. 8J). We stained the tissue with F4/80 (also known as ADGRE4), a macrophage marker, to measure inflammatory infiltration of macrophages. Anti-IL-6 Ab abrogated the anti-inflammatory function of LCG during muscle regeneration but had no effect in HCG conditions. Anti-TNFα Ab had no effect in LCG conditions, but significantly decreased inflammatory macrophage infiltration upon HCG (Fig. S8C,D). The results of H&E staining showed that anti-IL-6 Ab injection abrogated the beneficial effect of LCG on myogenesis, measured by myofiber CSA and number of central nuclei, but had no effect on HCG. By contrast, anti-TNFα Ab injection significantly reversed the deleterious effect of HCG (Fig. 8K-M). These data indicate that the gelatin–ROS–IL-6/TNFα cascade during skeletal muscle regeneration is valid *in vivo*.

## DISCUSSION

We have discovered that a modest injection of gelatin into injured skeletal muscle can aid the regeneration process. We also found pleiotropic effects of gelatin on myogenesis *in vitro* and *in vivo* mediated by the intracellular ROS–IL-6/TNFα signal cascade, which is reminiscent of hormesis in toxicology. These results suggest that gelatin as a functional biomaterial can be pro-regenerative during skeletal muscle regeneration, but its dose must be carefully controlled.

Gelatin is the natural product of collagen during ECM remodeling in skeletal muscle regeneration (MacQueen et al., 2019). Although many studies focused on the application of gelatin-based materials in muscle engineering, it is unclear whether gelatin influences the biological function of myoblasts and muscle regeneration. Our study has revealed the complex effects of gelatin on myogenesis *in vitro* and skeletal muscle regeneration *in vivo* through the ROS signaling pathway. The apparently paradoxical findings of gelatin can be explained within the framework of hormesis, defined as a bi-phasic dose response. The present work first illustrates the occurrence of hormesis in the biological function of ECM proteins and further indicates that this hormesis appears to be independent of biological models, *in vitro* cultured cells or *in vivo*. The therapeutic or toxic effect of gelatin on skeletal muscle regeneration is changeable in a dose-dependent manner. LCG plays a beneficial role in muscle regeneration, but HCG aggravates the muscle damage to delay the repair. The adverse effect of HCG on myogenesis might be attributed to the aging or growth arrest of cells, but the specific mechanism of HCG-induced damage needs further investigation. The safety profile of gelatin necessitates careful analysis of the risk/benefit balance prior to proposing clinical application.

As the hydrolysate of collagen, gelatin (non-fibrous without triple-helicity) is locally and temporally produced in skeletal muscle during inflammation and regeneration (MacQueen et al., 2019). It was reported that gelatin fibers artificially produced by spinning induced cell aggregation in rabbit skeletal muscle myoblasts and regulated aligned muscle tissue formation (MacQueen et al., 2019). Gelatin hydrogels promote the myogenesis of human muscle progenitor cells (Yi et al., 2017). This effect may be mediated by RGD (R, arginine; G, glycine; D, aspartate) domain interacting with integrin receptors (αvβ3, α5β1 and α7β1) at the cell surface (Elliott et al., 2005). It was shown that gelatin coating facilitates the adhesion and spread of C2C12 myoblasts through integrin receptors (αvβ3 and α5β1) (Davidenko et al., 2016). Integrin receptors influence F-actin assembly to regulate cell morphological changes through the FAK/Rho A/ROCK pathway (Wei et al., 2020). In this study, gelatin induced changes in cell morphology and migration, suggesting that integrin receptors might be the mediator of gelatin’s effects.

There is growing evidence supporting the effects of gelatin on ROS generation and redox function. Gelatin enhances ROS to induce the aggregation of peritoneal macrophages and the release of pro-inflammatory factors (Zhang et al., 2019). Gelatin facilitates the production of myofibroblasts from 3T3-L1 and C2C12 cells via ROS. Gómez-Guillén et al. reported that gelatin hydrolysates can protect mouse embryonic fibroblasts from UVB irradiation via the ROS pathway (Gómez-Guillén et al., 2011). Gelatin-derived peptides also exert antioxidant effects through multiple pathways, such as by inhibiting lipid peroxidation, scavenging free radicals and chelating transition metal ion (Gómez-Guillén et al., 2011). Peptides isolated from fish skin gelatin were shown to protect liver cells from oxidative damage by the ROS donor *t*BHP (Kim et al., 2001). These studies have indicated that gelatin exerts pleiotropic functions through ROS signal in different tissues.

We found that gelatin triggers ROS production both by the NADPH oxidase, NOX2, and mitochondrial respiratory complex I. Little is known about the function of gelatin in the NADPH system, except for one report showing that gelatin gel might increase the resistance of the NADPH-oxidoreductase enzyme system (Esimbekova et al., 2009). Collagen is shown to induce O_2_^−^ via regulating the activity of NOX at p47 (phox) (the organizer subunit) during platelet activation (Jang et al., 2015; Wang et al., 2015), but how gelatin affects the NADPH system remains to be elucidated. Similarly to HCG, gelatin nanoparticles have been reported to decrease the activities of SOD, GSH-PX and CAT during apoptosis of NCI-H460 lung cancer cells (Karthikeyan et al., 2015). Although gelatin influences the generation of ROS in different biological processes, the underlying mechanisms are not fully understood.

Skeletal muscle is an endocrine tissue as it produces and secretes cytokines, also known as myokines, growth factors and pro-inflammatory factors, which play important roles in muscle regeneration (Iizuka et al., 2014). ROS are related to the induction and accumulation of myokines such as IL-6 in myogenesis (Ost et al., 2016). IL-6 is a multifunctional cytokine, functioning not only as a pro-inflammatory factor but also as a myokine released from muscle in exercise or regeneration (Pedersen and Febbraio, 2012). It has been reported that the level of IL-6 in muscle can be transiently elevated 100-fold after exercise for 4 h (Belizário et al., 2016). IL-6 exerts a critical role in muscle homeostasis and diseases, and is related to hypertrophic muscle growth and myogenesis via regulating muscle stem cells (Madaro et al., 2018; Steyn et al., 2019). Our result that LCG promotes Pax7^+^ cell expansion is consistent with this finding. TNFα is a major pro-inflammatory cytokine that is increasingly expressed in damaged or dystrophic muscle (Erekat and Al-Jarrah, 2018). It was shown that a high level of TNFα might cause apoptosis of myoblasts and myocytes, thus causing muscle atrophy (Bhatnagar et al., 2010). Previous studies have shown that collagen I influences the migration and differentiation of myoblasts by regulating the release of IL-6 (Liu et al., 2020). Gelatin has also been shown to regulate the production and secretion of IL-6 and TNFα in differentiated U937 lymphoma cells (Zhao et al., 2018). In the present report, we showed that gelatin can induce IL-6 and TNFα in myoblasts at different concentrations.

In addition to TNFα, gelatin has also been shown to induce IL-1β and prostaglandin E_2_ in macrophages (Zhang et al., 2019). Kojima et al. reported that bovine bone gelatin stimulates the secretion of cytokines IL-6, MCP-1 and MIP-2 (also known as CXCL2) in murine adherent spleen cells to regulate cell proliferation (Kojima et al., 2005). However, how gelatin and ROS functions diverge through IL-6 and TNFα signaling in myoblasts remains unclear. One possibility is that the different amounts and species of ROS lead to activation of specific pathways. ROS and myokines have both been shown to promote muscle adaptation to exercise (Scheele et al., 2009). It was reported that the IL-6/STAT3 pathway participates in myoblast proliferation and macrophage migration during muscle regeneration (Zhang et al., 2013). The TNFα/NF-κB p65 pathway inhibits the myogenic differentiation of myoblasts, implicated as a mediator of muscle wasting (Langen et al., 2004). The versatile downstream myokines may further diversify cell adaptation behaviors to ECM and gelatin, warranting further investigation.

The present study has shown that a single injection of gelatin produces sustained reactions, such as ROS and IL-6/TNFα secretion, which were detectable at 2 weeks after injection. Several studies have reported similar results. Wang et al. described that the application of gelatin sponge contributes to wound healing after a 2-week transplantation (Wang et al., 2020). Gelatin hydrogels containing fibroblast growth factor and platelet-rich plasma enhanced angiogenesis of skeletal muscle after 1-week intramuscular injection (Matsui and Tabata, 2012). It was reported that an ultrasound-responsive gelatin complex containing a DNA plasmid enhanced the expression of genes in skeletal muscles after 5 days of intramuscular injection (Aoyama et al., 2002). The gelatin nanospheres loading miR-194 could promote skeletal muscle regeneration by promoting muscle differentiation and inhibiting ubiquitin ligase activity after 4 weeks of intramuscular injection (Zhang et al., 2021). Gelatin persists after 1-week injection into peripheral fat, and it can migrate into the entire episcleral space through the adipose orifice (Matsui and Tabata, 2012). These previous studies indicate the lasting presence of gelatin and/or gelatin effect. The degradation of gelatin is dependent on the activities of gelatinase, such as MMP-2 and MMP-9, which induce the degradation of collagen and gelatin under physiopathological conditions (Verma and Hansch, 2007). Studies have shown that CTX-induced skeletal muscle injury is accompanied by altered MMP activities (Gowda et al., 2006), and CTX III has been reported to inhibit MMP-2 and MMP-9 to reduce metastasis (Yen et al., 2013). We suspect that MMPs may be inhibited by CTX, which delays the degradation of gelatin. At the tissue level, we suspect that the interplay between SCs, immune cells (e.g. macrophages), ECM and myoblasts regulated by ROS and IL-6/TNFα crosstalk may have long-lasting effects over weeks.

Detailed investigation of ECM remodeling and intracellular signaling pathways for skeletal muscle regeneration has yet to be conducted. Given the physiological importance and promise of using multifunctional gelatin as a biomaterial in tissue engineering and regenerative medicine, better elucidation of such fundamental pathways is likely to give rise to therapeutic value.

## MATERIALS AND METHODS

### Animals

All experiments were performed according to P. R. China legislation on the care and use of laboratory animals and the criterion confirmed by the Institute for Experimental Animals at Shenyang Pharmaceutical University. C57BL/6 male mice at the age of 6-8 weeks, weighing 18-22 g, were obtained from Changsheng Biotechnology (Liaoning, China). Mice were maintained in a light (12 h light/12 h dark cycle)- and climate (temperature 23±0.5°C, humidity 55-65%)-controlled environment and fed a normal chow diet with water *ad libitum*.

### Skeletal muscle injury and gelatin injection

Ten-week-old C57BL/6 male mice were anesthetized and injected with PBS (Ctrl) or CTX (20 μl, 10 μM) in the hindlimb TA muscle. At 2 D.P.I., mice were injected with either saline or gelatin (20 μl, 5 and 20 mg/ml in saline) into injured TA muscle. The muscles were harvested at 7 and 14 D.P.I. for the analyses.

### Treatment with IL-6- and TNFα-neutralizing Abs

IL-6-neutralizing Ab (0.25 mg/kg, 25 μl), TNFα-neutralizing Ab (0.5 mg/kg, 25 μl) or saline was intramuscularly injected into uninjured, LCG or HCG treated-mice at 3 days post-CTX injury, and mice were sacrificed at 14 D.P.I. for analyses.

### Histological analysis

TA muscles were dissected from mice, embedded into OCT compound and quick-frozen by immersing in liquid nitrogen, then sectioned on a cryostat (AS-620; Shandon, Astmoor, UK) at a thickness of 10 μm. The 10% formalin-fixed muscle sections were further stained with Mayer's H&E (Sigma-Aldrich, St Louis, MO, USA). Immunofluorescence staining was also performed for subsequent further analysis. Tissue images were acquired on a fluorescence microscope (Nikon, Tokyo, Japan), and Image Composite Editor (Version 2.0.3.0) was used to make the collages presented in figures.

### Reagents

Primary Abs against MyoD (18943-1-AP) and MyoG (67082-1-Ig) were purchased from Proteintech (Wuhan, Hubei, China). The monoclonal neutralization Abs against IL-6 (AB-406-NA) and TNFα (AB-410-NA), and recombinant mouse proteins IL-6 and TNFα, were purchased from R&D Systems (Minneapolis, MN, USA). Fluorescein (FITC)-conjugated AffiniPure goat anti-mouse IgG (H+L) was obtained from KeyGEN Biotechnology (Nanjing, Jiangsu, China). DCFH-DA, NAC, *t*BHP, DPI and ROT were purchased from Sigma-Aldrich.

### Isolation and differentiation of MPMs

Primary myoblasts were isolated from 3-day-old C57/BL6 mice. The hindlimb muscles were aseptically dissected, minced mechanically and digested with 0.2% collagenase II (17101015, Invitrogen, Carlsbad, CA, USA) and 0.05% trypsin in PBS for 40 min at 37°C with slight agitation. Then, the mixture was filtered with a 200-mesh (75 μm) filter and centrifuged at 400 ***g*** for 5 min. Cells were resuspended in PBS, and separated in 20%/60% Percoll (65455-52-9, Solarbio, Beijing, China) by centrifugation at 800 ***g*** for 20 min. They were then resuspended in growth Ham's F10 medium (11550043, Gibco, Grand Island, NY, USA) containing 20% fetal bovine serum (FBS; 10099141, Gibco), 2.5 ng/ml basic FGF (HEGFP-0602, Cyagen, Suzhou, China) and 1% penicillin/streptomycin and were cultured on plates (Corning Inc., Corning, NY, USA) for 2 h; the non-adherent cells were then transferred to another dish. For inducing myogenic differentiation, the culture medium of cells at 90% confluence was replaced with Dulbecco's modified eagle medium (DMEM; high glucose, Gibco) containing 2% horse serum (26050070, Gibco). Cells were cultured in the differentiation medium for another 5 days until formation of myotubes.

### Cell culture and myogenic differentiation

C2C12 myoblast cells (American Type Culture Collection, Manassas, VA, USA) were cultured in DMEM supplemented with 10% FBS (Beijing Yuanheng Shengma Research Institution of Biotechnology, Beijing, China), 100 μg/ml streptomycin and 100 U/ml penicillin (growth medium), at 37°C and 5% CO_2_. For inducing myogenic differentiation, the culture medium was replaced with DMEM containing 2% horse serum (differential medium) when cells grew to 90% confluence (Gibco, NY, USA). Cells were cultured in differential medium for a further 5 days until the formation of myotubes was detected by positive staining of MyHC. Myotube quantifications were described as the myogenesis index and the fusion index. The myogenesis index is defined as the number of nuclei within MyHC-stained myocytes versus the total number of nuclei × 100%. The fusion index is defined as the nuclei number in myotubes with five or more nuclei versus the total number of nuclei in MyHC^+^ cells × 100% (Zhang et al., 2015).

### Preparation of gelatin-coated dishes

Gelatin was prepared in the Nippi Research Institute of Biomatrix (Toride, Ibaraki, Japan) and was diluted in 0.5 mM acetic acid to the indicated concentrations (5, 10, 20 mg/ml) and added to cell culture dishes (Corning Inc.), incubated for 4 h at 37°C, 5% CO_2_. Before plating cells, gelatin solution was discarded, and the remaining gelatin was rinsed off by PBS three times (Wang et al., 2016).

### Cell growth and migration detection

Cell growth was examined using a CCK-8 kit (Beyotime Biotechnology, Shanghai, China) according to the manufacturer's instructions. Briefly, C2C12 cells (8×10^3^ cells/well) were cultured for 24, 48 and 72 h on 96-well plates pre-coated with gelatin (100 μl/well). Then, CCK-8 reagents (10 μl) were added to each well, and cells were further incubated for 1 h at 37°C. The absorbance at 450 nm was recorded using a microplate reader (Multiskan MK3, Thermo Fisher Scientific, Shanghai, China).

Migrations were determined by cell-scratch and transwell cell migration assays as described previously (Liu et al., 2020, 2018c). For transwell assay, cells pre-cultured on gelatin-coated dishes were collected and plated into the upper insert of transwell plates with 8 μm pores (Costar, NY, USA) at a density of 6×10^4^ cells/well with serum-free DMEM for 6 h, which allowed cells to migrate across the filter pores. Then, cells were fixed with 4% paraformaldehyde (PFA) for 20 min and stained with 0.1% Crystal Violet for 30 min. The non-migrating cells on the upper surface were scraped, and the migration ratio of cells was examined by the absorption of Crystal Violet at 570 nm. For cell scratch, cells cultured on gelatin-coated dishes were scratched using 200 μl tips and then were refreshed with serum-free DMEM for 24 h. The migration ratio was determined by the gap between scratched wounds.

### Confocal fluorescence microscopic analysis

The indicated cells were fixed with PFA for 20 min and permeabilized with 0.15% Triton X-100 for 12 min. Then, the cells were blocked with PBS containing 10% FBS for 30 min. For MyHC and Desmin staining, cells were incubated with anti-MyHC Ab (MAB4470, R&D Systems; 1:50) or Desmin (AF5334, Affinity Biosciences, Cincinnati, OH, USA; 1:100) overnight at 4°C in PBS, and then treated with fluorescent secondary Ab (1:200) for 2 h at room temperature in the dark. After incubation with the secondary Ab, cells were stained with 4′,6-diamidino-2-phenylindole (DAPI; Beyotime Biotechnology; 1 μg/ml) for 10 min, and imaged with a confocal microscope (Nikon).

### Immunohistochemistry and immunofluorescence

TA muscle sections were fixed with 4% PFA and permeabilized with 0.2% Triton X-100 for 10 min in PBS. Then, sections were incubated with 3% H_2_O_2_ for 20 min to remove the endogenous peroxidase and blocked with 10% normal goat serum for 2 h at room temperature. Sections were incubated with primary Ab against eMyHC (F1.652, Developmental Studies Hybridoma Bank, Iowa City, IA, USA; 1:50), laminin (L9393, Sigma-Aldrich; 1:100), Pax7 (AF7584, Affinity Biosciences; 1:300), F4/80 (DF2789, Affinity Biosciences; 1:500) or MyoG (67082-1-Ig, Proteintech; 1:300) at 4°C overnight. For immunofluorescence, sections were treated with FITC- or TRITC-conjugated secondary Abs for 2 h and DAPI for 10 min to observe the muscle with a confocal microscope (Nikon). For immunohistochemistry, sections were incubated with biotinylated goat secondary Ab specific to the host species at room temperature for 1 h, and then visualized by DAB solution (MXB Biotechnologies, Fuzhou, China) for 5 min. The images were captured using a phase-contrast microscope.

### EdU staining

Cells cultured on gelatin-coated dishes for 24 h were incubated with EdU (C0071S, Beyotime Biotechnology; 10 μM) for 2 h, fixed in 4% PFA, permeabilized by 0.5% Triton X-100, and then detected according to the manufacturer's protocol with Hoechst 33342 for cell nuclei.

### Enzyme-linked immunosorbent assay (ELISA)

Cells (3.0×10^5^ cells/well) were cultured on gelatin-coated dishes until reaching 90% confluence. Then, cells and supernatants were harvested separately, and the cell number in each well was counted under a microscope. The amount of detected myokines was normalized to cell numbers in each well. TA muscles were minced by a grinder and crushed by ultrasonication in PBS (1:9). The homogenate was centrifuged at 800 ***g*** for 20 min, and supernatants were collected. Cytokines IL-6, MCP-1, CCL5, TNFα, IL-1β and IL-18 in cell cultures, and IL-6 and TNFα in muscle tissue, were examined using ELISA kits (DakeweBiotech, Shenzhen, China) following the manufacturer's protocols.

### Gene expression analysis

Total RNA was extracted using RNAiso plus reagent (TaKaRa, Tokyo, Japan) following the manufacturer's instructions. One microgram of RNA was used to synthesize the first strand of cDNA using a PrimeScriptTM RT-PCR Kit (TaKaRa). Quantitative PCR was conducted using PrimeScriptTMRT Master Mix (TaKaRa) on ABI 7500 Fast to quantify mRNA of *Myod* (accession number: NM_010866.1), *Myog* (accession number: NM_031189.2) and MyHC (*Myh1*) (accession number: NM_030679.1). Primer sequences are as follows: *Myod* forward, 5′-CATTCCAACCCACAGAACCT-3′; *Myod* reverse, 5′-CAAGCCCTGAGAGTCGTCTT-3′; *Myog* forward, 5′-CAATGCACTGGAGTTCGGT-3′; *Myog* reverse, 5′-GCCAGGTTGACATTGGATTG-3′; *Myh1* forward, 5′-CGCAAGAATGTTCTCAGGCT-3′; *Myh1* reverse, 5′-GCCAGGTTGACATTGGATT-3′; *Gapdh* forward, 5′-TCCCACTCTTCCACCTTC-3′; *Gapdh* reverse, 5′-CTGTAGCCGTATTCATTGTC-3′.

Relative expression of target genes was determined by comparing to *Gapdh* mRNA. The mRNA index of myogenic proteins was calculated and plotted as an integrated fold change of *Myod*, *Myog* and MyHC mRNA to control by using the scale function in R-package.

### Transfection of siRNA

Cells were transfected with siRNA targeting NOX2 and negative control using Lipofectamine 2000 (Invitrogen) according to the manufacturer's instructions. Then, the cells were incubated for another 48 h before subsequent experiments. The sequences used are as follows: si-NOX2-1: (sense) 5′-CAGTGTGTCGAAATCTGCTCTCCTT-3′, (antisense) 5′-AAGGAGAGCAGATTTCGACACACTG-3′; si-NOX2-2: (sense) 5′-CAGTGCGTGTTGCTCGACAAGGATT-3′, (antisense) 5′-AATCCTTGTCGAGCAACACGCACTG-3′; si-NC (negative control): (sense) 5′-TTCTCCGAACGTGTCACGTTT-3′, (antisense) 5′-ACGTGACACGTTCGGAGAATT-3′.

### Western blot analysis

The cells and supernatants were harvested to acquire protein samples using RIPA lysis buffer (Beyotime, Haimen, China) containing protease inhibitor cocktail (Solarbio, Beijing, China). TA muscles were minced and crushed by ultrasonication in RIPA lysis buffer (1:9). The homogenates were centrifuged at 13,780 ***g*** for 10 min, and supernatants were collected. Protein concentrations were examined by BCA (WLA004b, Wanleibio, Shenyang, China) according to the manufacturer's instructions. The proteins were separated by 10-12% SDS-PAGE. After electrophoresis, the proteins were transferred to Immobilon^®^-P Transfer Membrane (Millipore, Billerica, MA, USA) and blocked in 5% skin milk solution for 2 h. Then, the membranes were incubated with primary Abs overnight at 4°C. The next day, the horseradish-peroxidase-conjugated secondary Abs were added to the membranes, incubated for 3 h and developed with SuperSignal^®^ West Pico Chemiluminescent Substrate (Thermo Fisher Scientific, Rockford, IL, USA). Primary Abs against MyoD (18943-1-AP; 1:1000) and MyoG (67082-1-Ig; 1:1000) were purchased from Proteintech (Wuhan, Hubei, China). Primary Abs against β-actin (BF0198; 1:4000), GAPDH (AF7021; 1:8000), NOX2 (DF6520; 1:500) and NOX4 (DF6924; 1:500) were obtained from Affinity Biosciences. Ab against MyHC (MAB4470; 1:1000) was purchased from R&D Systems.

### Examination of ROS, enzyme activity and MDA

The indicated cells and supernatants were collected and lysed in RIPA buffer. TA muscles were minced and crushed by ultrasonication in pre-cooled saline (1:9). The homogenates were centrifuged at 13,780 ***g*** for 10 min, and supernatants were collected. Protein concentrations in lysates were quantified using a BCA kit (Solarbio, Beijing, China). The content of O_2_^−^ (A052-1-1), ·OH (A018-1-1) and H_2_O_2_ (A064-1-1) was examined using kits (Nanjing Jiancheng Bioengineering Institute, Nanjing, China) according to the manufacturer's instructions. The activities of SOD (A001-3-2), GSH-PX (A005-1-2) and CAT (A007-1-1), and the content of MDA (A003-1-2), were measured using kits (Nanjing Jiancheng Bioengineering Institute) according to the manufacturer's instructions.

### Measurement of ROS

ROS in the total intracellular and TA muscle were examined using DCFH-DA or MitoSox Red staining (Ye Sen, Shanghai, China). For intracellular and mitochondrial ROS, the indicated cells were incubated with 10 μM DCFH-DA or 5 μM MitoSOX Red at 37°C for 30 min, washed three times with PBS and analyzed using flow cytometry. To visualize mitochondrial ROS, the indicated cells were incubated with 100 nM MitoTracker Green FM and 5 μM MitoSOX Red at 37°C for 30 min (Ye Sen). The cells were then washed three times with PBS and fixed with PFA for 20 min. Then, they were permeabilized with 0.15% Triton X-100 for 12 min and blocked with 10% FBS for 30 min. Lastly, the cells were stained with DAPI for 10 min and imaged with a confocal microscope (Nikon). For TA muscle ROS, the tissue was minced, and digested with 0.2% collagenase II and 0.05% trypsin for 40 min to obtain single-cell suspension. The cells were resuspended with 10 μM DCFH-DA in PBS for 30 min after the centrifugation, followed by washing three times with PBS and analyzed using flow cytometry. For the DCFH-DA staining of TA muscle, sections were washed with PBS and incubated with DCFH-DA (10 μM) at 37°C for 30 min. Then, sections were washed with PBS and incubated with DAPI at room temperature for 10 min to visualize nuclei. The stained sections were observed with a confocal microscope (Nikon).

### Statistical analysis

All data were analyzed using Prism software (Version 6.01, GraphPad, La Jolla, CA, USA). Unpaired Student's *t*-test with Welch's correction and one-way ANOVA followed by post-hoc testing (Tukey's correction) were used to examine the comparisons between groups. *P*<0.05 was considered statistically significant, and data are shown as means±s.e.m.

## Supplementary Material

Supplementary information
