# Supplementary material for: Bi-phasic effect of gelatin in myogenesis and skeletal muscle regeneration
Source: Dis Model Mech. 2021 Dec 24;14(12):dmm049290. doi: 10.1242/dmm.049290 (PMC8713995; doi:10.1242/dmm.049290)
Supplement: Supplementary information [file dmm-14-049290-s1.pdf]

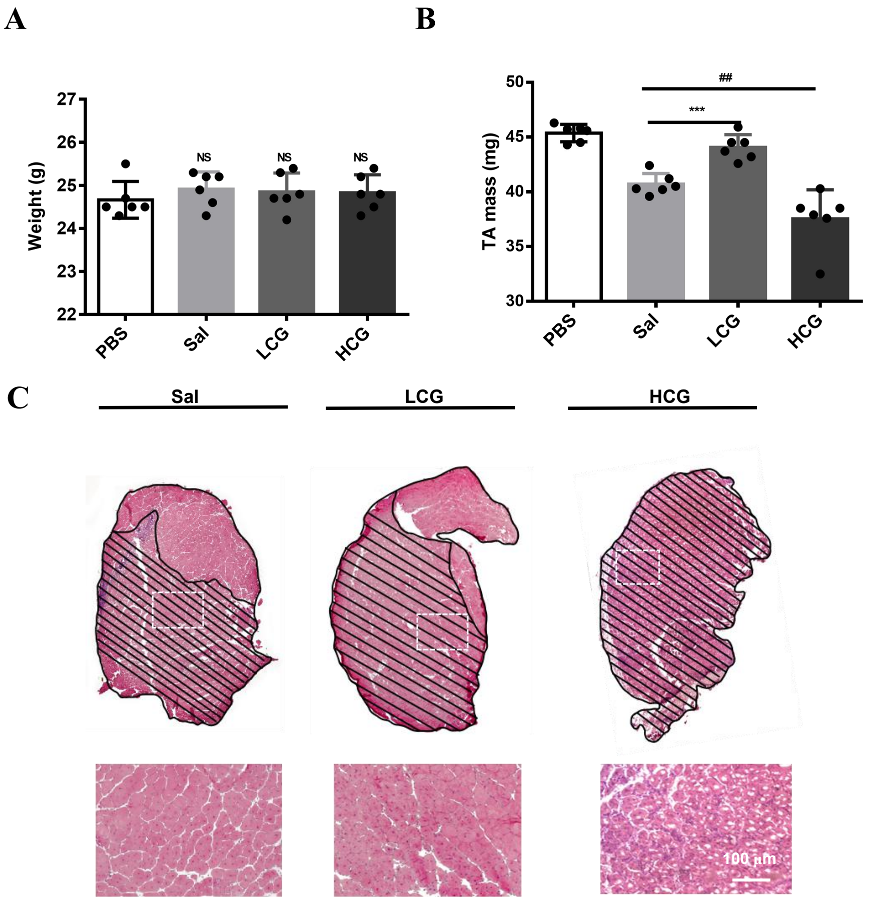

Fig. S1. Bell-shaped recovery response of skeletal muscle to gelatin. A. The animal body weight and **B.** TA muscle mass at 14 D.P.I. of uninjured animals (PBS), and injured animals treated with vehicle (Sal), LCG, and HCG (*n*=6). **C.** Whole-section view of H&E staining of 14 D.P.I. TA muscle. Hatched areas highlight damage and regeneration with centralized nuclei caused by intramuscular CTX treatment. Scale bar, 100 µm. Significance was determined by unpaired two-tailed Student's *t*-test with Welch's correction. $^{\#\#}P < 0.01$; $^{***}P < 0.001$; NS, not significant. Data are mean ± s.e.m.

Disease Models & Mechanisms • Supplementary information

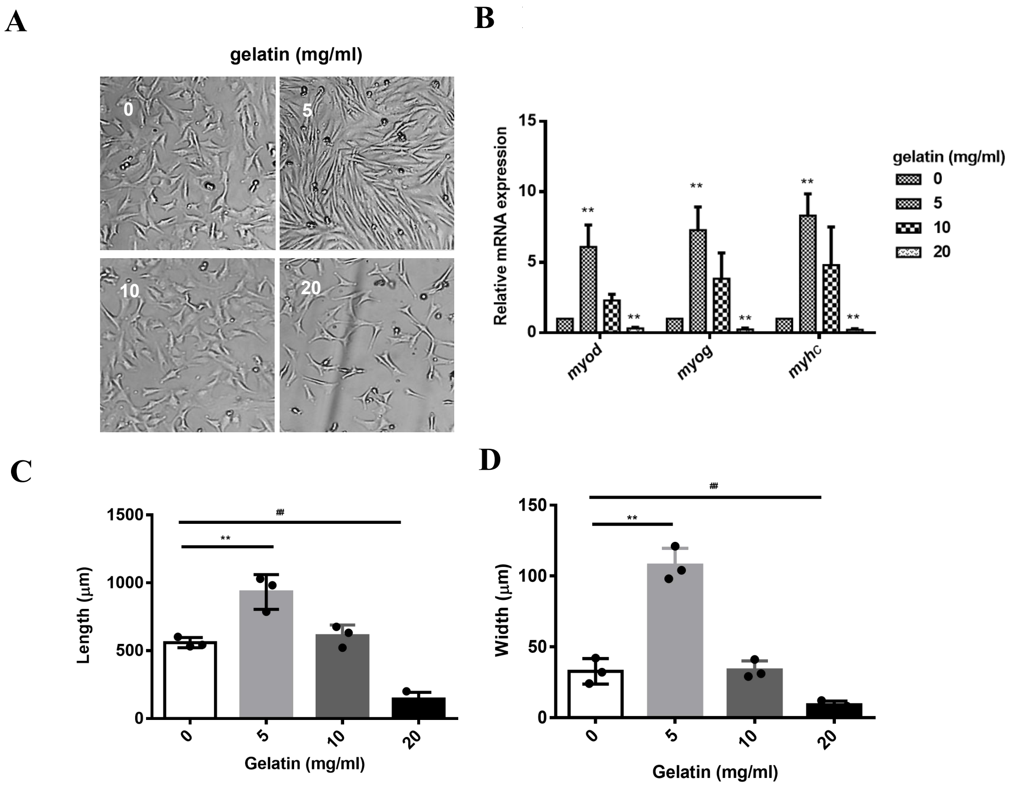

Fig. S2. Bell-shaped responses of myogenesis to gelatin. Cells were cultured on gelatin-coated dishes for 24 h. **A.** phase-contrast images of C2C12 cells. **B** The mRNA level of MyoD, MyoG and MyHC. β-Actin is used as a loading control. **C-D.** Width and length of myotubes were calculated using Image-Pro Plus software. Significance was determined by unpaired two-tailed Student's *t*-test with Welch's correction.. $^{\#\#,}$ ***P* < 0.01. (*n*=3, mean ± s.e.m).

Disease Models & Mechanisms • Supplementary information

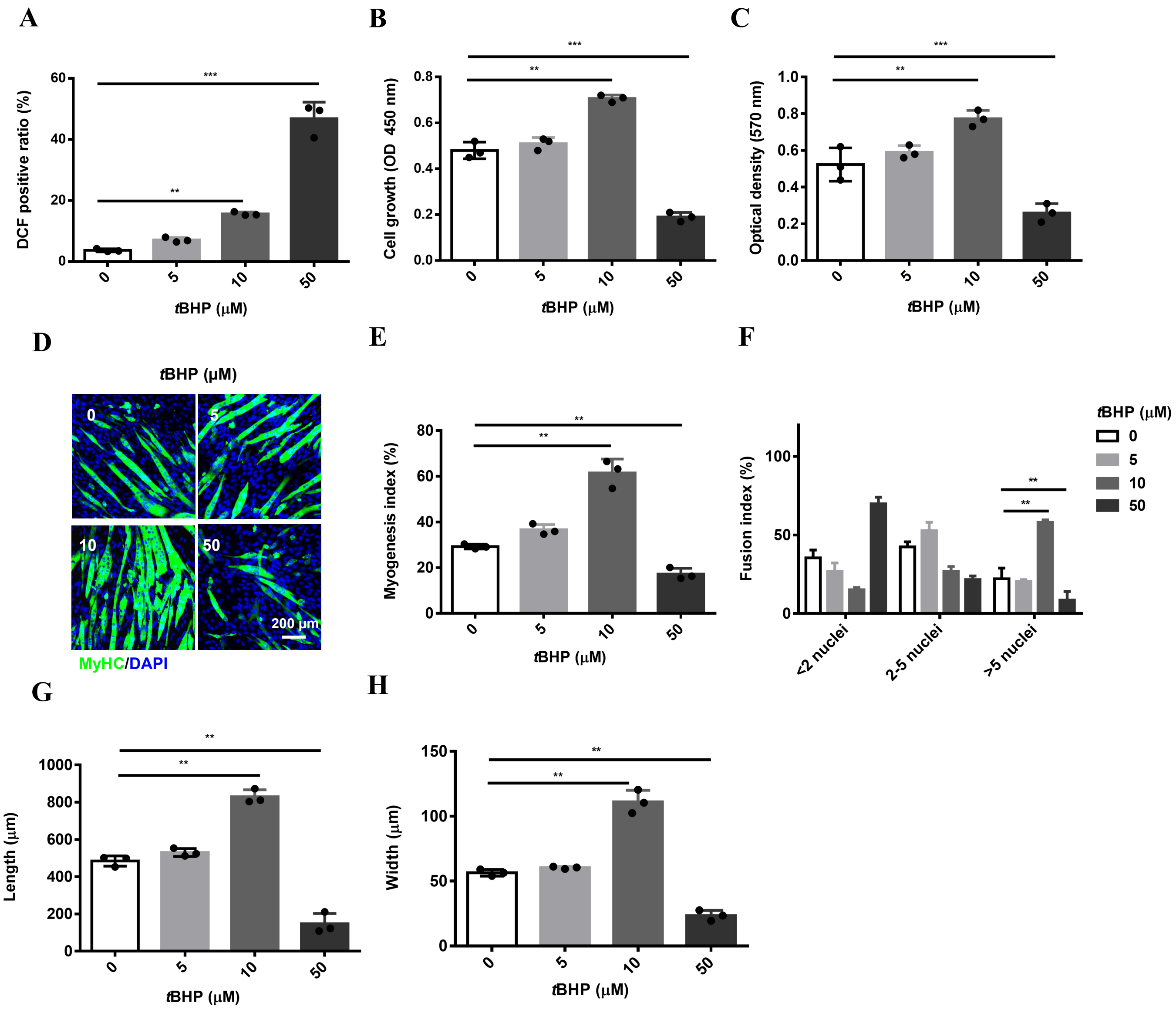

Fig. S3. Dual effect of tBHP on myogenesis. A. ROS levels were examined by DCFH-DA staining and flow cytometry in **c**ells treated with different concentrations of *t*BHP. **B.** The growth and **C**. migration of cells were examined by CCK-8 kits and Transwell assay, respectively. **D.** Confocal images of myogenic differentiation of cells stained with MyHC antibody. Scale bar, 200 µm. **E-H.** Myogenesis index, myotube fusion, the width and length of myotubes were calculated using Image-Pro Plus software. Significance was determined by unpaired two-tailed Student's *t*-test with Welch's correction. $**P < 0.01$; $***P < 0.001$. (n=3. mean ± s.e.m).

Disease Models & Mechanisms • Supplementary information

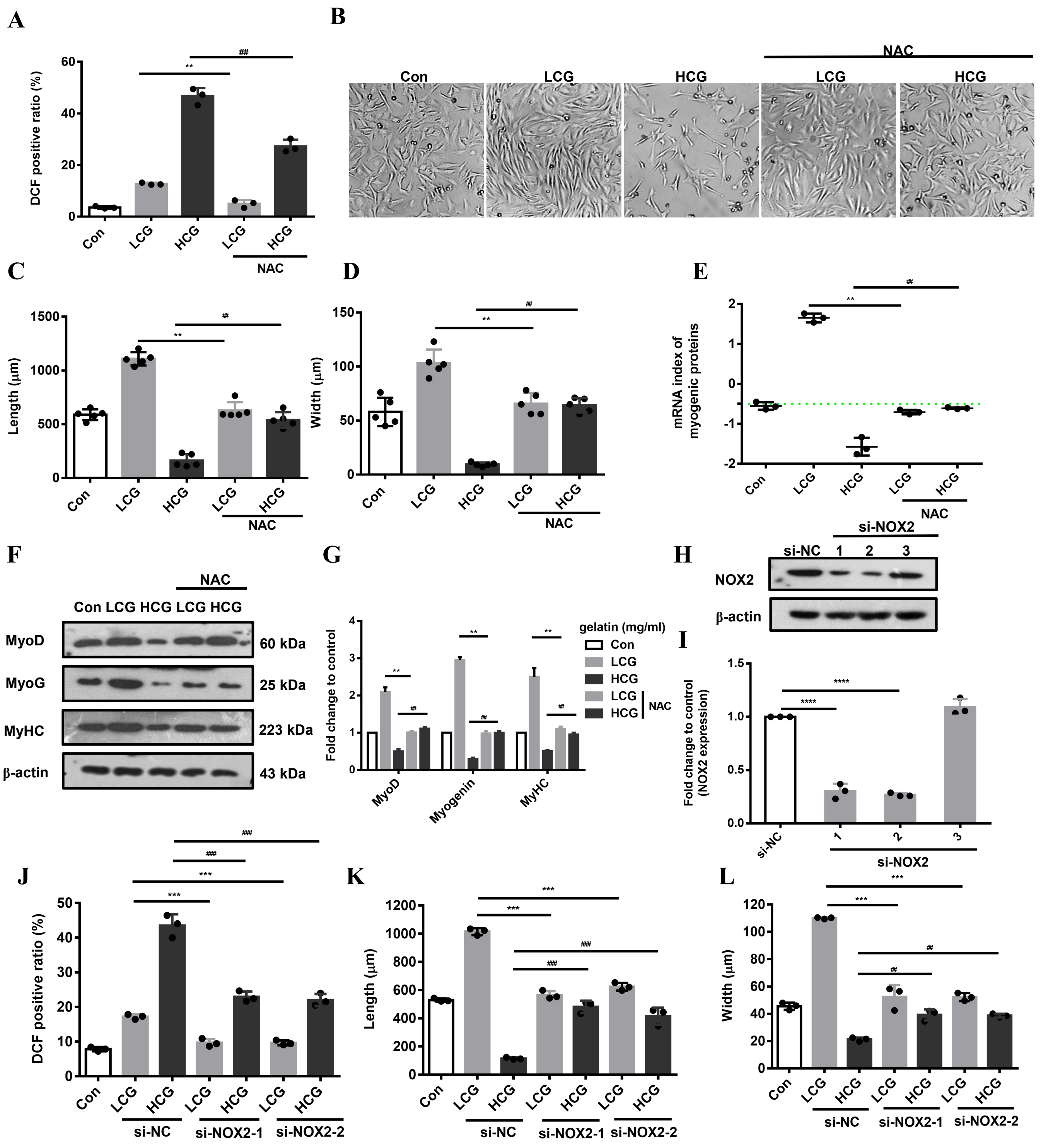

Fig. S4. ROS signaling is required for the bi-phasic role of gelatin. A. ROS levels in cells treated with ROS scavenger NAC (8 mM). **B**. The phase-contrast images of C2C12 cells treated with NAC on LCG or HCG-coated dishes. **C**. The width and **D.** length of myotubes were calculated by Image-Pro Plus software (n=5). **E-G.** The mRNA and protein levels of MyoD, MyoG and MyHC were examined by RT-PCR and western blot. **H-I.** Knockdown efficiency of NOX2 by siRNA was examined by western blots (si-NC: negative control siRNA). β-Actin is used as a loading control. **J.** ROS levels in cells transfected with si-NOX2 or si-NC. **K.** The width and **L.** length of myotubes were calculated by Image-Pro Plus software. Significance was determined by one-way ANOVA with Tukey's post-hoc test. ##, **$P < 0.01$; ###, ***$P < 0.001$. Data are mean ± s.e.m.

Disease Models & Mechanisms • Supplementary information

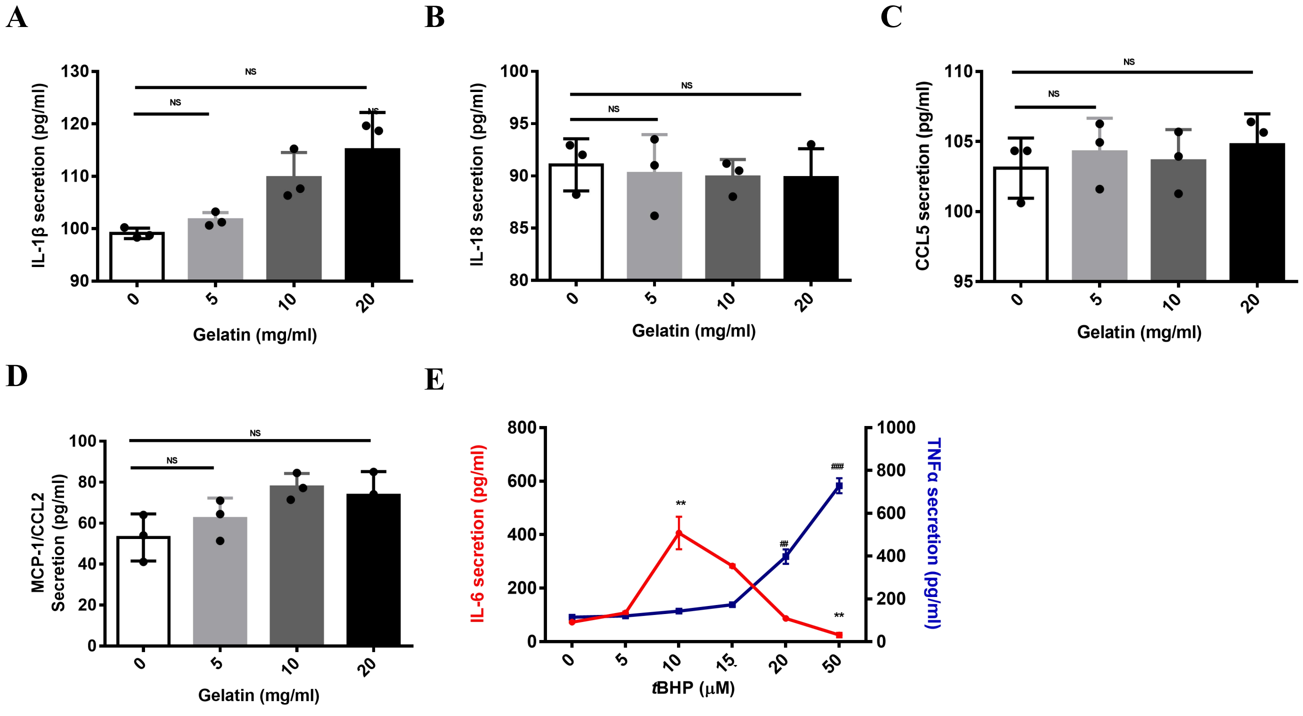

Fig. S5. ROS mediate the release of IL-6 and TNFα from myoblast cells. A-D. The release of IL-1β, IL-18, CCL5 and CCL2/MCP-1 examined by ELISA kits. **E.** The release of IL-6 and TNFα from cells treated with different concentrations of *t*BHP. Significance was determined by unpaired two-tailed Student's *t*-test with Welch's correction. $^{\#\#,}$ $^{**}P < 0.01$; $^{\#\#\#}P < 0.001$; NS, not significant. (n=3. mean ± s.e.m).

Disease Models & Mechanisms • Supplementary information

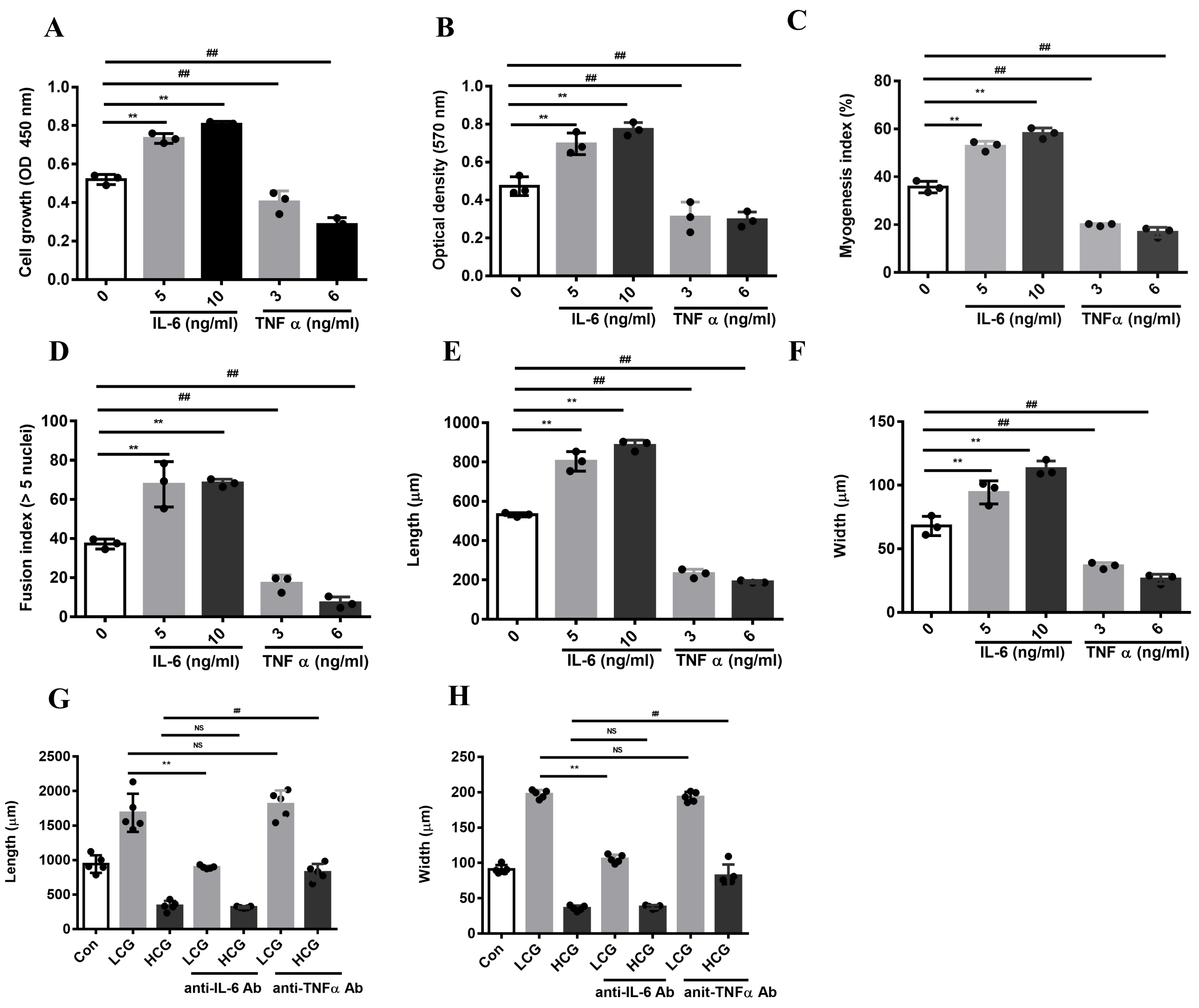

Fig. S6. IL-6 and TNFα are required for the biphasic role of gelatin. A. The growth and **B**. migration of cells treated with mouse recombinant IL-6 proteins (5 and 10 ng/ml) and TNFα proteins (3 and 6 ng/ml) were examined by CCK-8 kits and Transwell assay, respectively. **C**. Myogenesis index, **D**. myotube fusion, **E**. the width and **F**. length of myotubes were calculated using Image-Pro Plus software. **G**. The width and **H**. length of myotubes were calculated using Image-Pro Plus software (*n*=5). Significance was determined by unpaired two-tailed Student's *t*-test with Welch's correction (Fig. S6A-F) and one-way ANOVA with Tukey's post-hoc test (Fig. 4G-H). $^{\#\#,}$ $^{**}P < 0.01$. NS, not significant. Data are mean ± s.e.m.

Disease Models & Mechanisms • Supplementary information

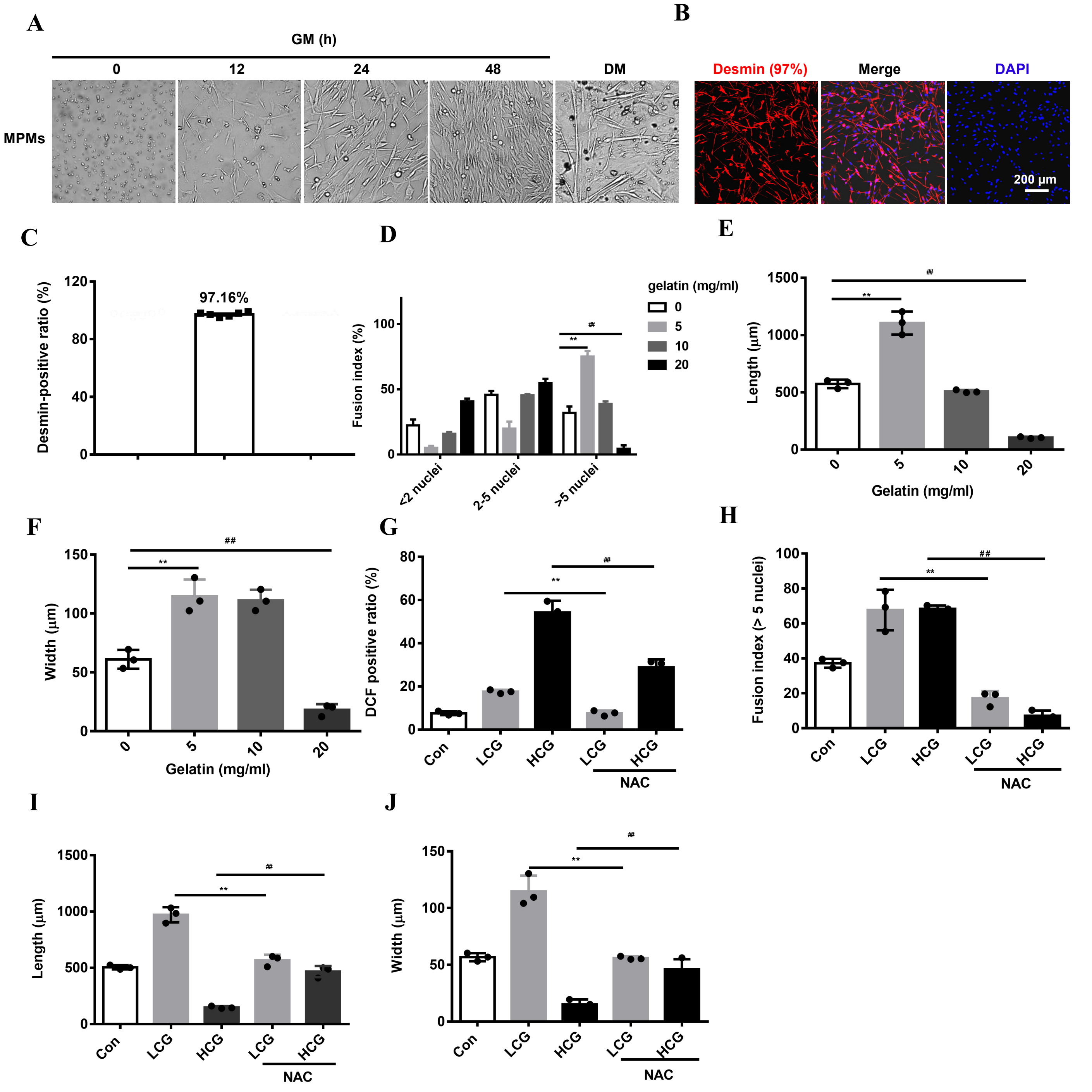

Fig. S7. Dual regulation of gelatin on myogenesis via ROS in mouse primary myoblasts (MPMs). A. Phase-contrast images of cultured MPMs (GM: growth medium; DM: differentiation medium) **B.** Immunofluorescence images of Desmin (MPM marker protein, red) and DAPI (blue). **C.** Quantification of the percentage of Desmin-positive cells. Scale bar, 200 μm. **D.** The myotube fusion, **E**. the length and **F**. width of myotubes were calculated using Image-Pro Plus software. **G.** ROS levels in NAC-treated. **H.** The fusion, **I.** length and **J**. width of myotubes were calculated using Image-Pro Plus software. Significance was determined by unpaired two-tailed Student's t-test with Welch's correction (Fig. S7D-F) and one-way ANOVA with Tukey's post-hoc test (Fig. 4G-J). ##, ***P* < 0.01. (n=3. mean ± s.e.m).

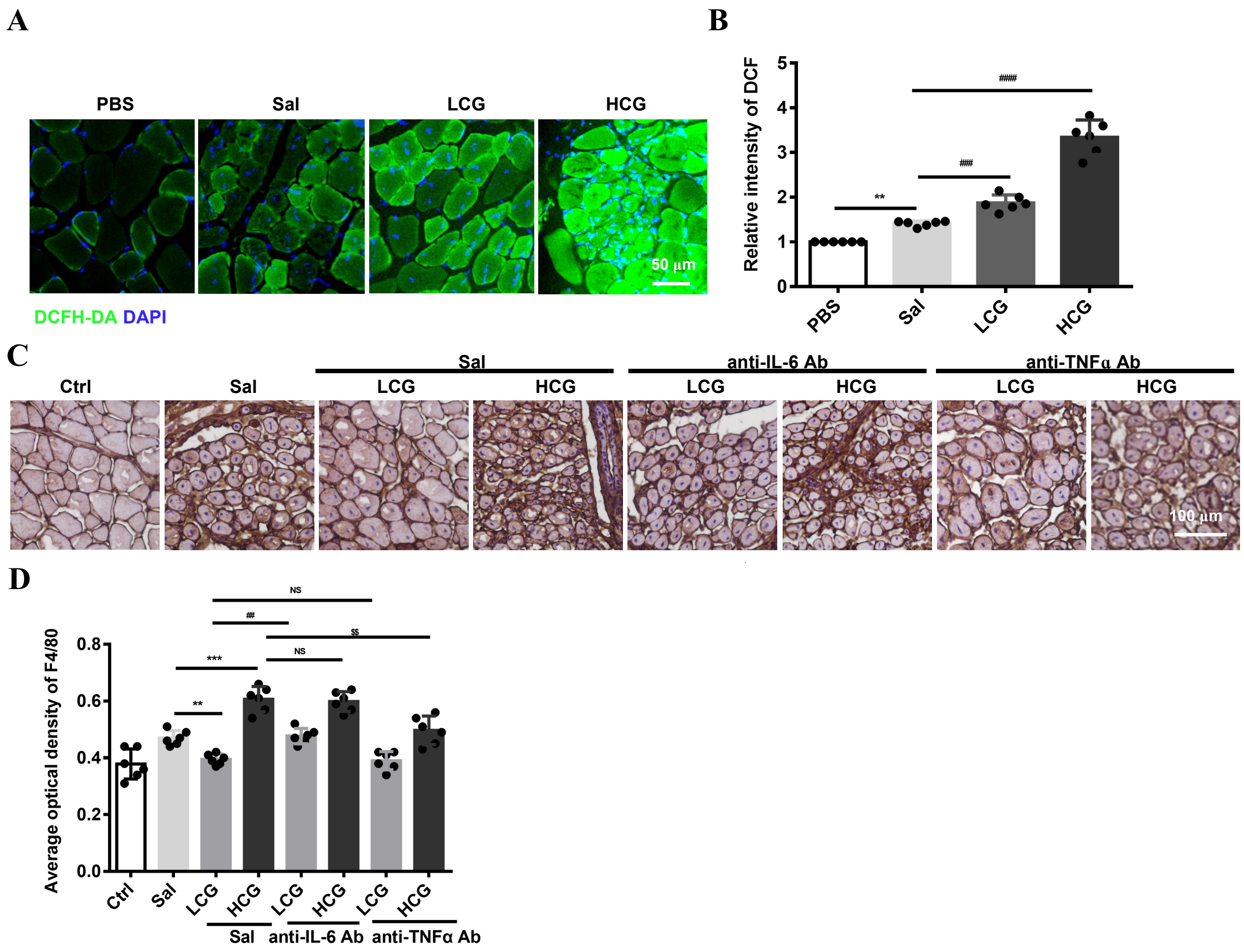

Fig. S8. Gelatin influences the muscle regeneration via ROS/IL-6/TNFα crosstalk *in vivo*. **A.** ROS were detected in TA muscle with DCFH-DA (10 µM, green) and DAPI stains nuclei (blue). Scale bar, 50 µm. **B**. Quantification of DCF immunofluorescence intensity (*n*=6). **C-D**. IHC staining with F4/80, macrophage marker for TA muscle of mice injected with anti-IL-6 Ab or anti-TNFα Ab at 3 D.P.I. Sal, saline. Scale bar, 100 µm. The mean optical density of F4/80 was calculated using Image J software (*n*=6). Significance was determined by one-way ANOVA with Tukey's post-hoc test. $^{\#\#,}$ **, $^{\$\$}P < 0.01$; $^{\#\#\#,}$ $^{***}P < 0.001$; NS, not significant. Data are mean ± s.e.m.

Disease Models & Mechanisms • Supplementary information
